# May microbial ecological baseline exist in continental groundwater?

**DOI:** 10.1186/s40168-023-01572-4

**Published:** 2023-07-19

**Authors:** Sining Zhong, Shungui Zhou, Shufeng Liu, Jiawen Wang, Chenyuan Dang, Qian Chen, Jinyun Hu, Shanqing Yang, Chunfang Deng, Wenpeng Li, Juan Liu, Alistair G. L. Borthwick, Jinren Ni

**Affiliations:** 1 College of Environmental Sciences and Engineering, Peking University; Key Laboratory of Water and Sediment Sciences, Ministry of Education, No. 5 Yiheyuan Road, Beijing, 100871 People’s Republic of China; 2 State Environmental Protection Key Laboratory of All Material Fluxes in River Ecosystems, Beijing, 100871 People’s Republic of China; 3 Fujian Agriculture and Forestry University, College of Resources and Environment, Fujian Provincial Key Laboratory of Soil Environment Health and Regulation, Fuzhou, 350002 People’s Republic of China; 4 State Key Laboratory of Plateau Ecology and Agriculture, Qinghai University, Xining, 810016 People’s Republic of China; 5 Center for Groundwater Monitoring, China Institute of Geo-environmental Monitoring, Beijing, 100081 People’s Republic of China; 6 School of Engineering, Computing and Mathematics, University of Plymouth, Drake Circus, Plymouth, PL8 4AA UK

**Keywords:** GMEB, Bacterial community, Keystone taxa, Deterministic processes, Groundwater

## Abstract

**Background:**

Microbes constitute almost the entire biological community in subsurface groundwater and play an important role in ecological evolution and global biogeochemical cycles. Ecological baseline as a fundamental reference with less human interference has been investigated in surface ecosystems such as soils, rivers, and ocean, but the existence of groundwater microbial ecological baseline (GMEB) is still an open question so far.

**Results:**

Based on high-throughput sequencing information derived from national monitoring of 733 newly constructed wells, we find that bacterial communities in pristine groundwater exhibit a significant lateral diversity gradient and gradually approach the topsoil microbial latitudinal diversity gradient with decreasing burial depth of phreatic water. Among 74 phyla dominated by *Proteobacteria* in groundwater, *Patescibacteria* act as keystone taxa that harmonize microbes in shallower aquifers and accelerate decline in bacterial diversity with increasing well-depth. Decreasing habitat niche breadth with increasing well-depth suggests a general change in the relationship among key microbes from closer cooperation in shallow to stronger competition in deep groundwater. Unlike surface-water microbes, microbial communities in pristine groundwater are predominantly shaped by deterministic processes, potentially associated with nutrient sequestration under dark and anoxic environments in aquifers.

**Conclusions:**

By unveiling the biogeographic patterns and mechanisms controlling the community assembly of microbes in pristine groundwater throughout China, we firstly confirm the existence of GMEB in shallower aquifers and propose Groundwater Microbial Community Index (GMCI) to evaluate anthropogenic impact, which highlights the importance of GMEB in groundwater water security and health diagnosis.

Video Abstract

**Supplementary Information:**

The online version contains supplementary material available at 10.1186/s40168-023-01572-4.

## Background

Groundwater, the world’s largest available store of freshwater resource, provides more than two billion people with drinking water and supplies approximately 40% of global irrigation [1]. Groundwater is vital to global biogeochemical cycles [2, 3]. As the most ancient and diverse life form on Earth, microbes comprise almost the sole ecological community found in groundwater [4, 5]. Over billions of years, groundwater microbes have participated in the metabolism of key elements such as carbon, nitrogen, sulfur, phosphorus, and various metals and thereby have influenced the biogeochemistry of subsurface and even surface ecosystems [6, 7]. Compared with the surface environment, aquifer ecosystems are harsh habitats for biological survival due to their being devoid of photosynthesis, oxygen, and readily available organic carbon [2, 8] and so offer ideal targets for the study of microbial ecology, evolution, and environmental adaptation [9, 10]. In the past decade, the tree of life has significantly expanded owing to the discovery of vast previously uncharacterized and uncultured microbial populations in aquifers [11–13]. For example, Brown et al. [11] newly defined >35 candidate phyla radiation (CPR), also known as *Patescibacteria*, by reconstructing 789 draft genomes from groundwater samples. The superphylum *Patescibacteria* has received extensive attentions, given its unique features of ultra-small cell size, small genome size, and lack of CRISPR viral defense, which brings new understandings on life of microbes in extreme environments [12, 14]. Different assemblages of *Patescibacteria* organisms are key to turning the globally relevant subsurface biogeochemical cycles [15, 16].

The ecological baseline delineates the original state of ecosystem attributes such as environmental parameters, biological composition, and service functions and could be applied to the design of operational monitoring programs that quantify ecosystem change in response to anthropogenic disturbance and contamination [17, 18]. Ecological baselines of soil, river, and ocean ecosystems established based on macro-organisms (e.g., fishes [19] and invertebrates [20]) have demonstrated that a return to the nearly original state could be expected upon the baselines being correctly determined and human interference being effectively controlled. Nowadays, groundwater is facing dual global threats to its water quality and quantity globally [21], and so an improved understanding is urgently needed of groundwater geochemistry and ecology in order to assess anthropogenic impact. Previous indices developed for groundwater ecological assessment, such as the groundwater quality index (WQI) [22], have invariably overlooked the significance of groundwater microbes. Meanwhile, the ubiquity, strong adaptability, and dispersal abilities of groundwater microbes have led to controversy as to whether or not microbial elements should be included in establishing the groundwater ecological baseline [23]. Thanks to the encouraging progresses in advanced technologies, such as new generation high-throughput sequencing [24], which provide tremendous opportunity to uncover the mysterious world of microbes and enable us to explore the groundwater microbial ecological baseline (GMEB).

With the rapid development of high-throughput sequencing, numerous studies have established that microbes exhibit obvious microbial biogeographic patterns in a wide variety of natural ecosystems, including terrestrial [25] and marine [26] systems. However, previous studies concerning groundwater ecosystems have been mostly limited to small scale, for example, contaminated areas [27], typical basins [28], and special geological zones [29], and so are unable to provide a holistic view of GMEB at large scale. Meanwhile, understanding of the mechanisms that govern microbial community assembly is crucial for predicting the response of ecosystems to human activity. Several investigators have indicated that microbial biogeographic patterns are controlled by deterministic processes, including abiotic and biotic factors [27, 30, 31]. Such deterministic processes increase the predictability of microbial communities, providing theoretical support for the presence of a microbial ecological baseline. Other researchers have stressed the important roles of ecological drift, dispersal limit, and even historical contingency in community assembly [32, 33]. Noting the significant habitat differentiation of complex heterogeneous environments in the subsurface, niche differentiation appears to offer a sensible ecological interpretation of variations in microbial diversity and composition [34, 35].

**Fig. 1:**
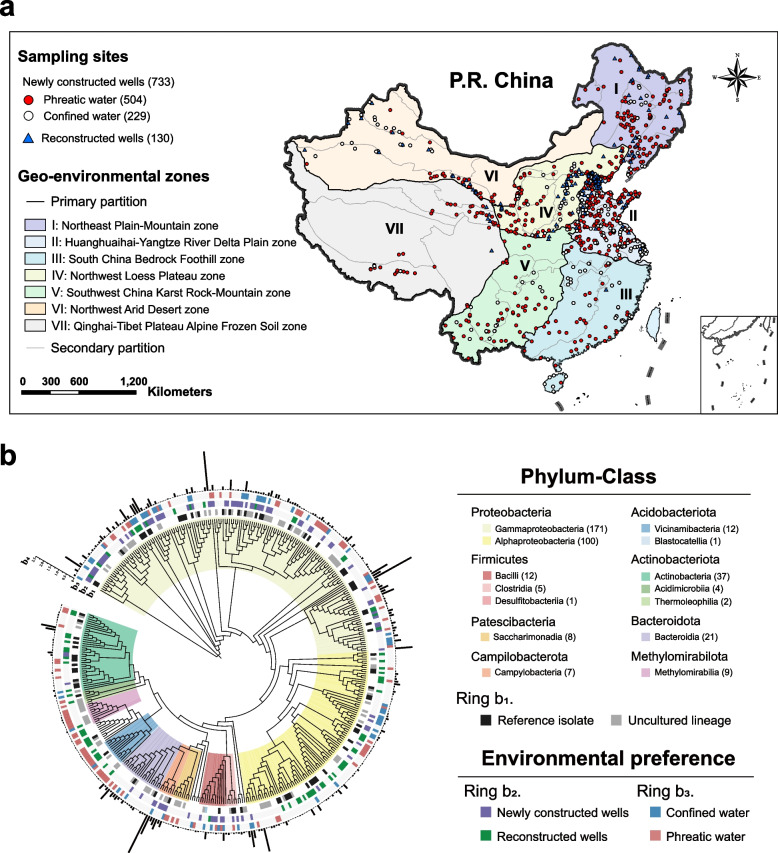
The atlas of dominant microbes in continental groundwater. **a** 863 sampling sites distributed throughout China. Groundwater samples collected from 733 newly constructed and 130 reconstructed wells are marked by circles and triangles. For newly constructed wells, red and white circles represent phreatic and confined groundwater samples. The background is a composite of seven geo-environmental zones. **b** Phylogenetic tree of core taxa in groundwater. The colors in the innermost ring indicate taxonomic information on core taxa at class level. On ring b_1_, black indicates a representative strain matched at the ≥ 97% similarity level, and gray indicates taxa identified as having uncultured lineage. The colors on rings b_2_ and b_3_ denote environmental preference. The histogram (b_4_) in the outermost ring displays average relative abundance

Considering the severe scarcity of baseline data concerning the groundwater microbial ecosystem, we implemented a national monitoring campaign covering 733 newly constructed and 130 reconstructed wells across China (Fig. 1a) and established a unique microbial dataset, which enables us to address the following major questions: (1) Does GMEB exist at continental scale? (2) What are the lateral and vertical patterns of baseline microbial communities in different geo-environments? (3) What are the dominators and keystone taxa in pristine groundwater? (4) Could the principal processes of community assembly be beneficial in shaping the GMEB? (5) Whether there is a good index to assess the anthropogenic impact on groundwater based on the GMEB?

## Materials and methods

### Study area and sample collection

As the largest country in Asia, China has abundant groundwater resources distributed across various climatic belts and geo-environmental zones and is ideal for exploring microbial communities in groundwater at continental scale. We obtained groundwater samples from 733 newly constructed wells and 130 reconstructed wells. In the newly constructed wells, sampling commenced immediately after exposure of groundwater to the external environment, thus providing first-hand samples useful as a baseline of groundwater microbes throughout China. Sampling from reconstructed wells enabled comparison with groundwater microbial communities in newly constructed wells, including 504 phreatic and 229 confined wells. The monitoring wells were distributed across seven geo-environmental zones covering 31 provinces in China (Fig. 1a, Table S1 and S2). The sampling campaign occupied a wide geographical space extending from 18.3° N to 52.0° N and from 76.1° E to 133.5° E. We particularly focused on areas facing groundwater problems, such as the Beijing-Tianjin-Hebei region where the groundwater has experienced severe overexploitation and salinization.

Prior to sampling, groundwater in a given monitoring well was abstracted at a controlled discharge below 100 mL/min using a submersible sampling pump. Outflow water quality indicators (pH, electrical conductivity, oxidation-reduction potential, and turbidity) were measured using a portable tester (AP-800, Aquaread Ltd.) at intervals ranging from 5 to 15 min until water quality stabilized over three consecutive measurements (≤ ±10%). More than 3000 L of groundwater were drained from each sampling site and filtered by hollow fiber membranes to enrich microbial cells (Toray, 0.01 μm). The hollow fiber membranes were transported with dry ice to designated laboratories and stored at −80 ℃.

Groundwater samples were collected in 5-L sterile PET bottles for physicochemical content analysis. Prior to analysis, the samples were transported to the laboratory within 12 h and stored at −4 °C. According to the standard methods prescribed by the Ministry of Ecology and Environment of China, an array of physicochemical parameters, including total dissolved solids (TDS), chemical oxygen demand (COD_Mn_), ammonium nitrogen (NH_4_^+^-N), and nitrate nitrogen (NO_3_^-^-N), were determined. Key metal elements (e.g., sodium (Na), potassium (K), calcium (Ca), and magnesium (Mg)) were measured by ICP-MS (Thermo Fisher Scientific, USA). Bicarbonate (HCO_3_^-^) and carbonate (CO_3_^2-^ ) were measured using potentiometric titration, and fluoride (F^-^), chloride (Cl^-^), and sulfate (SO_4_^2-^) were determined by ion chromatography (Thermo Fisher Scientific, USA). All physicochemical parameters were normalized using Min-Max standardization.

### DNA extraction and bioinformatics analysis

The substances captured by the hollow fiber membranes were dissolved in ultrapure water by ultra-sonication, then filtered through 0.22-μm polycarbonate membranes (Millipore, USA). Genomic DNA was extracted using the MoBio PowerSoil® kit (MoBio Laboratories, Carlsbad, CA, USA) according to manufacturer protocols. DNA quantity and quality (Table S3) were determined using a NanoDrop Spectrophotometer (NanoDrop Technologies Inc., Wilmington, DE, USA). Polymerase chain reaction (PCR) was used to amplify the V3-V4 hypervariable region of the bacterial 16S rRNA gene (3 min at 95 °C, followed by 29 cycles at 95 °C for 30 s, 55 °C for 30 s, and 72°C for 45 s, and concluding with a final extension step at 72 °C for 10 min). Primers used for bacterial 16S rRNA gene PCR amplification were 338F (5′ -ACTCCTACGGGAGGCAGCAG-3′) and 806R (5′ -GGACTACHVGGGTWTCTAAT-3′) [36]. Sequencing was performed by Shanghai Majorbio Bio-pharm Technology Company Ltd. (Shanghai, China).

DNA sequences were quality-filtered on the Majorbio Cloud Platform (https://cloud.majorbio.com/) using QIIME v1.9.1 [37]. Operational taxonomic units (OTUs) were clustered with 97% similarity cutoff using UPARSE (version 7.1) [38], and chimeric sequences were identified and removed using UCHIME. A representative sequence of each OTU was selected for taxonomic assignment. Bacterial OTUs were assigned by the RDP classifier [39] against the SILVA 16S rRNA database (http://www.arb-silva.de/). A confidence threshold of 70% was used to analyze the taxonomy for all OTUs. OTUs identified at the level of phylum, family, order, class, genus, and species were 86.7%, 80.4%, 61.6%, 38.3%, 23.9%, and 8.5%, respectively.

### Statistical analysis

#### Identification of the core microbial taxa (OTUs)

The core microbial taxa in groundwater were identified from the huge, unique datasets established for this study, following two criteria [40]. Firstly, we identified the most abundant OTUs based on average relative abundance < 0.01%. Secondly, only ubiquitous OTUs occurring in > 50% of the total samples were considered. To identify the environmental preference of each core microbial taxa between newly constructed and reconstructed wells, the Wilcoxon rank-sum test was applied using the wilcox.test function in “stats” package in R version 3.6.1 (https://www.r-project.org/). A similar test was conducted for core taxa between confined and phreatic groundwater in newly constructed wells. Sequences of core OTUs were compared with those archived in the National Center for Biotechnology Information (NCBI) nucleotide database, using the Basic Local Alignment Search Tool (BLAST) to obtain a more accurate phylogenetic tree. The closest sequences and selected reference sequences were aligned using ClustalW software. After alignment, gaps were trimmed with the trimAl tool (threshold = 0.2). The phylogenetic tree was constructed by the MEGA 7.0 tool using a neighbor-joining algorithm with a bootstrap test of 1000 replicates and maximum composite likelihood model [41] and visualized using an online Interactive Tree Of Life server (https://itol.embl.de/).

#### Alpha and beta diversity

The OTU table for subsequent comparative analysis was rarefied to the same sequencing depth (23,976 sequences per sample). Alpha diversity was quantified using MOTHUR [42]. Taxonomic and phylogenetic diversities were measured using the Shannon diversity index and Faith’s phylogenetic diversity. Linear and polynomial regression fits were constructed using the nlme R package. Non-metric multidimensional scaling (NMDS) was used to visualize the dissimilarity of beta diversity based on the Bray–Curtis distance. One-way analysis of variance (ANOVA) and analysis of similarity (ANOSIM) were calculated to test the significance of differences in community diversity and structures among specific groups using the “aov” and “anosim” functions in vegan R package, respectively. Distance–decay relationships (DDRs) were calculated as the slopes of linear least-squares regressions for relationships between the natural logarithm of geographic distance and the natural logarithm of Bray–Curtis community similarity.

#### Identification of biomarker

Linear discriminant analysis effect size (LEfSe) was used with Wilcoxon and Kruskal–Wallis tests to discover high-dimensional biomarkers and explain taxa differences over varying well-depth ranges and geo-environmental zones. The LEfSe biomarker detection was performed in QIIME using the logarithmic LDA threshold > 3.5 and the statistical parameters of *P* < 0.05.

#### Network analysis

Co-occurrence network analysis at genus level was performed to investigate the complex interactions among microbial communities for different well-depth ranges (0–20, 20–40, 40–60, 60–80, and > 80 m). Firstly, rare genera with relative abundance of < 0.01% were removed. Secondly, all possible Spearman’s correlation coefficients between two genera were calculated. Then, species pairs with strong (Spearman’s |*r*| > 0.6) and significant (FDR-adjusted *P* < 0.001) correlations were selected to filter the data for reduced network complexity. Co-occurrence network visualization and modular analysis were conducted using the interactive platform Gephi (http://gephi.github.io/). The topology of networks (including average degree, average path length, clustering coefficient, graph density, and modularity) and node-level topological features (including degree, betweenness, and closeness centrality) were characterized using the igraph R package. Higher average degree, clustering coefficient, graph density, and lower average path lengths suggest a more connected co-occurrence network [43]. High mean degree, high closeness centrality, and low betweenness centrality were jointly used as thresholds for identifying keystone taxa [44].

#### Niche breadth

The niche breadth (B) index was estimated according to the formula [45]:

$${B}_{j}=1/{\sum }_{i=1}^{N}{{P}_{ij}}^{2}$$

where *B*_*j*_ indicates the niche breadth of species *j*; *P*_*ij*_ is the proportion of species* j* present in habitat *i*. Species with a higher B-value are considered to be habitat generalists whereas species with a lower B-value are habitat specialists. Habitat niche breadths and mean niche breadths (OTUs) at community level were calculated as the summation and average of B-values of all taxa occurring in a single community [46].

#### Ecological models

Fitness of zero-sum multinomial (ZSM), pre-emption, broken stick, log-normal, Zipf, and Zipf–Mandlebrot models were employed to confirm whether niche or neutral processes determined the community assembly within a sample. Akaike information criterion (AIC) values for the pre-emption, broken stick, log-normal, Zipf, and Zipf–Mandlebrot models were calculated using the “radfit” function in the vegan R package. The AIC value of ZSM model was determined using Tetame [47]. All models were compared based on their AIC values, with a lower AIC value indicating a better fit of the model to the sample [48]. The normalized stochasticity ratio (NST) was used to estimate ecological stochasticity of community assembly, with 50% taken as the boundary point between more deterministic (< 50%) and more stochastic (> 50%) assemblies [49, 50]. NST values for microbial communities in different groundwater samples were calculated according to taxonomic and phylogenetic metrics using the NST R package.

#### Influence of environmental variables

Variation partitioning analysis (VPA) was conducted to address the relative roles of geographical and environmental factors and their combined effect on community variations, based on the Bray–Curtis distance [51]. The Mantel test (999 permutations) was performed to examine the correlation between environmental variables and community structures. Environmental variables with variance inflation factors >10 were removed to ensure the absence of multicollinearity among environmental variables. Constrained correspondence analysis (CCA) of beta diversity with environmental variables was undertaken to investigate community distribution. VPA, Mantel test, and CCA were carried out using the vegan R package. Pearson and Spearman correlation analyses were performed using SPSS software (IBM Corporation, USA), and the corresponding heatmap plotted using the ggplots R package. Detailed information on the grouping variables and statistical hypothesis for the analytical methods used in the study is provided in Table S4. Bonferroni correction p.adjust methods in the stats R package were used to provide strong control of the family-wise error rate.

#### Groundwater Microbial Community Index (GMCI)

GMCI described the characteristic of microbial community by means of an integrated variable, analogous to and modified from the Invertebrate Community Index (ICI) [52] and Rapid Assessment Approach [20]. The procedure was as follows: (1) Construction of baseline data. Selection of the baseline sites as reference data must follow two principles, i.e., no-disturbance (or minimal level of anthropogenic interference) and relatively similar type of habitat to the monitoring site. (2) Selection of a subset of microbial indicators. Microbial diversity, dominators, key species, and biomarkers of pristine groundwater were selected as initial indicators. Any species with an occurrence rate less than 20% or average relative abundance less than 0.5% was excluded. (3) Observation and expectation ratio (O/E ratio) of microbial indicators was determined for the test sites. The 60% baseline and test samples were randomly selected to estimate the expectation value and set the alarm O/E ratio of each indicator, while each of the remaining samples was judged as to whether it had experienced strong anthropogenic interference by comparing its O/E ratio with the alarm O/E ratio. Indicators with low identified accuracy rate (accurate identified number / actual number of reconstructed wells) and high error rate (error identified number / actual number of newly constructed wells) would be eliminated. (4) Integration and calculation of GMCI. Multiple reliable indicators with weights and scores were integrated into a single index namely GMCI. An alarm threshold value of GMCI = 1.0 was used to evaluate the status of each observed microbial community in groundwater, and the identified accuracy and error rate of anthropogenic interference then calculated.

## Results

### Profiles of microbial communities in groundwater

A total of 97,569 OTUs (operational taxonomic units sharing ≥ 97% sequence similarity), belonging to 74 phyla and 1703 genera, were obtained by high-throughput sequencing of groundwater samples acquired throughout China. Proteobacteria was the most abundant phylum (20.5% of the total OTUs and 52.1% of the total 16S rRNA sequences), followed by *Bacteroidota*, *Campilobacterota*, *Patescibacteria*, *Actinobacteriota*, *Firmicutes*, *Desulfobacterota*, *Chloroflexi*, *Acidobacteriota*, *Nitrospirota*, *Methylomirabilota*, and *Verrucomicrobiota* (Additional file 2: Fig. S1).

Similar to microbial communities in other systems [40, 53], the species rank abundance distribution of groundwater microbes at national scale presented a typical peak-and-tail distribution (Additional file 2: Fig. S2), in which 1186 most abundant OTUs accounted for 74.9% of the total abundance, whereas 93.0% OTUs comprised regionally rare OTUs with a mean relative abundance of < 0.001% [54]. Based on previous studies [40], we defined the core microbial taxa as OTUs of occurrence frequency > 50% and mean relative abundance > 0.01%. About 0.42% of OTUs (411) constituted the microbial core community in groundwater, accounting for 53.8% of the total abundance (Fig. 1b). Less than 20% of the core OTUs matched an available reference genome at > 97% similarity level and 23.4% were uncultivated lineages. Most of the core OTUs belonged to *Proteobacteria* (*Gammaproteobacteria* and *Alphaproteobacteria*), *Actinobacteriota*, *Bacteroidota*, and *Firmicutes*. It is likely that these core taxa share certain phenotypic traits and/or life-history strategies to adapt to harsh subterranean habitats. For example, the genus *Pseudomonas* contained the most abundant and the largest number of core phylotypes in groundwater, which proved to have low nutritional requirements and a high diversity of energy metabolisms [55].

### Lateral and vertical pattern of baseline microbes

**Fig. 2:**
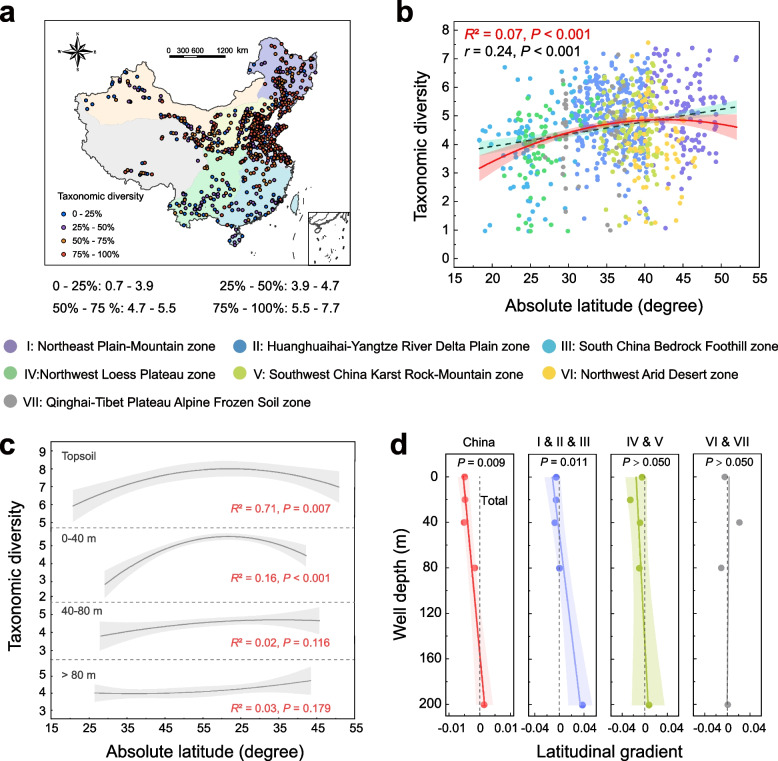
Biogeographic patterns of groundwater baseline microbes in China. **a** Spatial distribution of groundwater microbial diversity across seven geo-environmental zones. **b** Microbial latitudinal diversity gradient (LDG) in groundwater. Red solid and black dashed lines show polynomial and linear fits based on ordinary least square regression, with the shaded area representing 95% confidence intervals. Values of the adjusted *R*^2^ of the polynomial fits and Pearson’s *r* of the linear fits are provided. **c** Comparison of LDG pattern in three well-depth ranges of phreatic water with that on the topsoil. **d** Vertical trend of LDG pattern in eastern (zone I, II, and III), middle (zone IV and V), and western (zone VI and VII) China. Quadratic coefficients of polynomial fits of LDG are used to represent their variation rate in varying well-depth ranges

Biogeographic patterns can provide important perspectives by which to understand ecological and evolutionary processes in a natural ecosystem [23]. Here we used Shannon’s diversity index and Faith’s phylogenetic diversity (PD) to derive biogeographic patterns of microbial alpha diversity in groundwater from 733 newly constructed wells across China. The taxonomic and phylogenetic diversities of groundwater microbes exhibited similar biogeographic patterns (Pearson’s coefficient: *r* = 0.85, *P* < 0.001), peaking at mid-latitudes (around 40° N, Fig. 2a, 2b) with a clear increasing trend from west to east of China (Additional file 2: Fig. S3). Microbial diversity across the seven geo-environmental zones exhibited significant discrepancy (one-way ANOVA test: *P* < 0.001) in phreatic water, highest in the Huanghuaihai-Yangtze River Delta Plain zone (II) and lowest in the South China Bedrock Foothill zone (III) (Additional file 2: Fig. S4). According to previous studies on the age-depth relationship in groundwater [56], phreatic water could be further classified into several levels in terms of the range of well depth (e.g., 0–40, 40–80, and > 80 m). As the well-depth range decreased from > 80 to 0–40 m, the latitudinal diversity gradient (LDG) in shallower groundwater (*R*^2^ = 0.16, *P* < 0.001) approached the topsoil LDG pattern (Additional file 1: Table S5 and Additional file 2: Fig. 2c), and the vertical change gradient was especially obvious in eastern China (zone I, II, and III, Fig. 2d).

The distance–decay relationship (DDR) is regarded as a fundamental pattern in ecology [53, 57]. The community similarity of groundwater microbes decreased significantly as geographical distance increased (Mantel *r* = 0.17, *P* < 0.001). M icrobial communities between varying geo-environments displayed steeper DDR slopes (Additional file 2: Fig. S5, slope = −0.21) than those within individual geo-environmental zones (slope = −0.10), suggesting an apparent influence of regional hydrogeological factors on microbial communities in groundwater. This finding was further confirmed by ANOSIM test at the OTUs level (*R*_ANOSIM_ = 0.27, *P* < 0.001).

Given that the vertical layering of strata is known to be unique and complex [2], we explored the relationship between microbial communities and placing depth of wells. In comparison to more productive systems (e.g., topsoil) [25, 58], microbial diversity in groundwater was much lower and exhibited a declining trend with increasing burial depth under varying geo-environments (Additional file 2: Fig. S6a and S7). This vertical trend was especially evident in phreatic water (Pearson’s coefficient: *r* = 0.41, *P* < 0.001), compared with the irregular variation of microbial diversity in confined water (*P* > 0.05). Non-metric multidimensional scaling (NMDS) analysis showed an obvious variation in microbial composition at OTUs level with well depth in phreatic water (Additional file 2: Fig. S6b), as confirmed by strong correlation between the second NMDS and well depth (*r* = −0.46, *P* < 0.001). Microbial communities in shallower phreatic water exhibited steeper DDR slope (0–40 m: slope= −0.18, Mantel *r* = 0.24, *P* < 0.001) and significantly higher β diversity (*P* < 0.001) than in deeper phreatic water (>80 m: slope= −0.02, Mantel *r* = 0.08, *P* > 0.05) (Additional file 2: Fig. S8).

### Biomarkers for depth-based microbial baselines in varying geo-environments

**Fig. 3:**
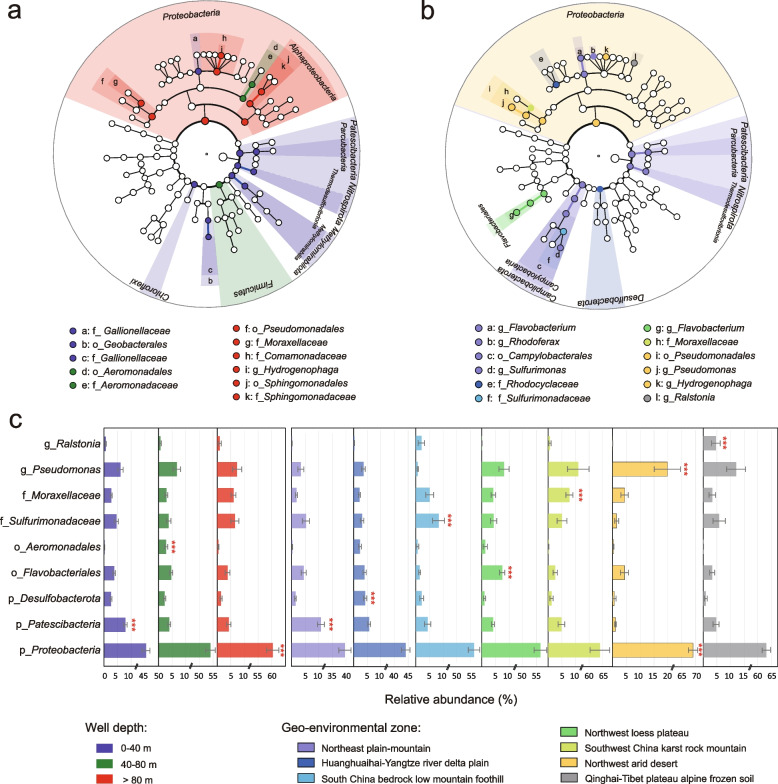
Biomarkers of varying groundwater samples. LEfSe cladogram showing biomarkers of **a** three well-depth ranges and **b** varying geo-environmental zones. Abundant taxa with average relative abundance of ≥ 0.5% are assigned to kingdom (innermost), phylum, class, order, family, and genus (outermost). Each biomarker is colored by its environmental preferences. **c** Spatial distribution of representative biomarkers for depth-based microbial baselines in varying geo-environments

To better understand the spatial heterogeneity of groundwater baseline microbial communities, we investigated the groundwater biomarkers in varying well-depth ranges (Fig. 3a) and geo-environmental zones (Fig. 3b and Fig. S9). Vertically, *Patescibacteria*, *Nitrospirota*, *Chloroflexi*, and *Methylomirabilota* preferred to occur in shallower groundwater (0–40 m), *Firmicutes* was more likely to appear in groundwater in the medium well-depth range (40–80 m), while *Proteobacteria* favored deeper groundwater (>80 m) and was the only phylum whose relative abundance increased significantly with well depth (Additional file 2: Fig. S10, *r* = 0.47, *P* < 0.001). Laterally, we provided the most representative biomarkers of each geo-environments. For example, genus *Ralstonia* could serve as a groundwater biomarker to distinguish from microbial communities in other geo-environmental regions, considering their much higher abundance in Qinghai-Tibet Plateau Alpine Frozen Soil zone (Fig. 3c).

As a superphylum of prevalent concern in recent years [14, 16], *Patescibacteria* was observed in more than 99.1% of groundwater samples, comprising 19.9% of the total OTUs (only second to *Proteobacteria*) and 5.7% of the total sequences (Additional file 2: Fig. S1). Relative abundance of *Patescibacteria* peaked in the Northeast Plain-Mountain zone (biomarker, 10.7±1.3%) and troughed in the Northwest Arid Desert zone (1.1±0.3%), mainly owing to habitat preferences of class *Parcubacteria* and *ABY1* (Additional file 2: Fig. S11b). *Patescibacteria* presented the most significant declining trend in relative abundance with increasing well depth in phreatic water (Additional file 2: Fig. S10, slope = −0.36, *r* = −0.55, *P* < 0.001) and exhibited a positive correlation with groundwater microbial diversity (Additional file 2: Fig. S12, *r* = 0.56, *P* < 0.001). In general, the vertical variation in dominant taxa appeared to weaken at lower taxonomy levels (e.g., class, order, family, and genus) (Additional file 1: Table S6), confirming previous claims that distributed randomness was greater among similar functional taxa and niche differentiation was stronger for a local community [59]. However, certain classes of *Patescibacteria*, notably *Parcubacteria*, *Microgenomatia*, *Gracilibacteria*, and *Berkelbacteria*, exhibited significant declines in relative abundance with increasing well depth (Additional file 2: Fig S11c).

### Coexistent patterns of baseline microbes

**Fig. 4:**
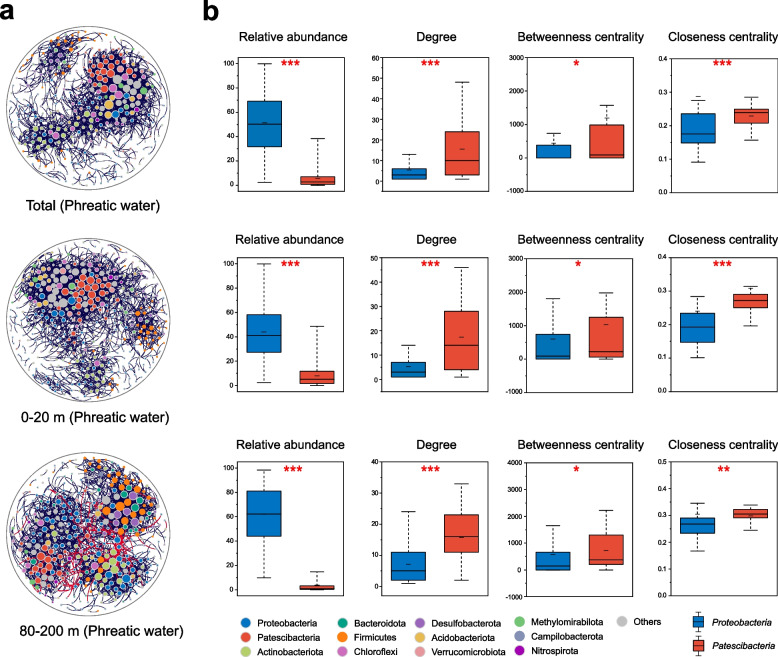
Coexistence patterns of baseline microbes. **a** Co-occurrence networks of microbial community at genus level (average relative abundance > 0.01%) for phreatic water samples. Each node represents one genus, and each edge represents a strong and significant correlation between two genera (Spearman’s |*r*| > 0.6 with FDR-adjusted *P* < 0.001). The size of each node is proportional to the degree, and the phyla of nodes are labelled in distinct colors. Black and red edges indicate positive and negative relationships. **b** Comparisons of relative abundance and node-level topological features (degree, betweenness centrality, and closeness centrality) between *Proteobacteria* and *Patescibacteria.* *0.01 < *P* < 0.05, **0.001 < *P* < 0.01, and ***P < 0.001

Microbial coexistent patterns in groundwater were further investigated through the establishment of co-occurrence networks based on microbial correlation relationships (Spearman’s |*r*| > 0.6 and FDR-adjusted *P* < 0.001) for several well-depth ranges (Fig. 4a). Microbes in deeper groundwater exhibited stronger interconnectivity than in shallower groundwater, characterized by higher average degree, clustering coefficient, and graph density, but lower average path length of subnetwork [43] (Additional file 1: Table S7). Positive and negative interactions in a co-occurrence network have previously been found to reflect potential mutualistic and antagonistic relationships among microbes [60]. Significant negative correlation was found only in deeper groundwater (6.02% negative edges for well depths > 80 m) possibly due to stronger competition among interspecies in deeper groundwater, whereas mutualism or commensalism were more likely to occur in shallower groundwater.

**Fig. 5:**
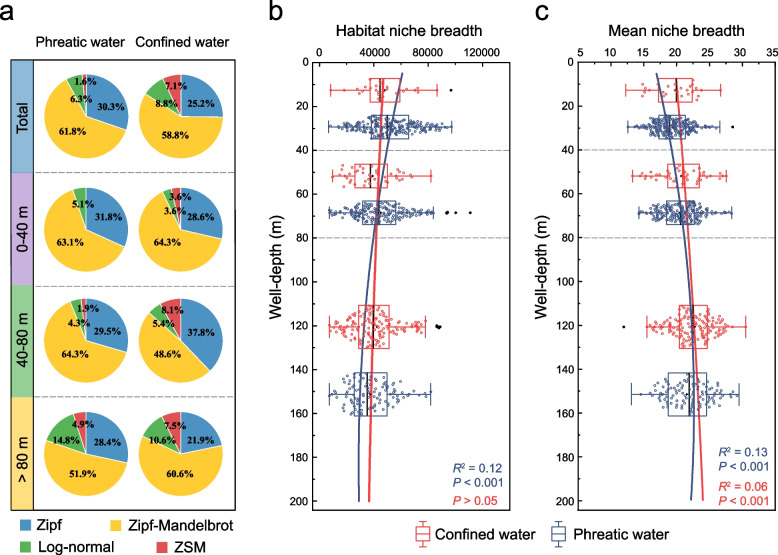
Deterministic community assembly of groundwater baseline microbes. **a** Proportions of samples fitted to pre-emption, broken stick, log-normal, Zipf, Zipf-Mandlebrot, and ZSM models at varying well-depth ranges (total, 0–40, 40–80, and > 80 m) in phreatic and confined water. ZSM was a neutral-based model, whereas the other models were niche-based. **b**, **c** Variations in habitat niche breadth and mean niche breadth (OTUs) of each sample with well-depth. Boxplots illustrate habitat niche breadth and mean niche breadth in phreatic (blue) and confined (red) water for varying well-depth ranges (0–40, 40–80, and > 80 m). Blue and red lines display the polynomial regression of niche breadth against well depth in phreatic and confined water

Node-level topological metrics such as degree, closeness centrality, and betweenness centrality can be used to identify keystone taxa [44]. In Fig. 5, most nodes in networks belonged to *Proteobacteria* whose relative abundance tended to increase with increasing burial depth. However, the degree and closeness centrality of *Proteobacteria* members were significantly lower than that of *Patescibacteria* (*P* < 0.01), implying a greater importance of *Patescibacteria* in maintaining structure and function of microbial communities in phreatic water. The keystone taxa largely belonged to the class *ABY1* and *Gracilibacteria* in shallower groundwater, with both having close connections with the taxa of *Proteobacteria*, *Chloroflexi*, *Dependentiae*, and *Verrucomicrobiota*. Whilst those in deeper groundwater (> 80 m) seemed more diverse, with the majority of taxa being capable of adapting to extreme environmental conditions or subsistence on persistent organic pollutants, such as *Sphingomonas* which is capable of degrading polycyclic aromatic hydrocarbons [61].

### Groundwater microbial ecological baselines supported by deterministic processes

To provide supporting evidence for GMEB, we assessed community assembly processes using several ecological models. Under the Akaike Information Criterion (AIC), we preliminarily confirmed the existence of GMEB by revealing the bacterial community assembly that was dominantly shaped by deterministic processes (Fig. 4a), with an exception of only 3.0% samples fitted to the ZSM model (neutral processes) [62]. This finding was further evidenced by the lower normalized stochasticity ratios [50] (NST < 50%) of community assembly based on taxonomic (average 29.62%) and phylogenetic metrics (average 32.54%) (Additional file 1: Table S8). Moreover, community-level habitat and OTU-level mean niche breadths were used to examine the variation in groundwater microbial diversity with burial depth. In phreatic water, habitat niche breadths were higher than those in confined water (*P* < 0.001) and exhibited an obvious declining trend with increasing burial depth (Pearson’s coefficient: *r* = −0.35, *P* < 0.001; polynomial fit:* R*^2^ = 0.12, *P* < 0.001) (Fig. 5b), further confirmed the increased competition among microbes for survival resource and space in deeper groundwater. Conversely, the mean niche breadths in phreatic water were significantly lower than in confined water (*P* < 0.001) and demonstrated a strongly positive correlation with well depth (Pearson’s coefficient: *r* = 0.28, *P* < 0.001; polynomial fit:* R*^2^ = 0.13, *P* < 0.001) (Fig. 5c), suggesting the significance of niche differentiation in shaping groundwater microbial ecological baseline pattern.

We performed variance partition analysis (VPA) based on Bray–Curtis similarity to evaluate the relative importance of environmental selection in groundwater microbial community assembly. Overall, the environmental variables provided a much more detailed picture of the spatial variation of the microbial community, particularly in shallower phreatic water (0–40 m, 15.27%, Additional file 2: Fig. S8b). Among the 58 parameters considered, the Mantel test suggested a relatively higher correlation between microbial structures and chemical oxygen demand (COD), nanganese (Mn), and bicarbonate (HCO_3_^-^) in groundwater (Additional file 2: Fig. S13). Canonical correspondence analysis (CCA) further indicated that geochemical signatures represented by Na^+^, K^+^, Cl^-^, and HCO_3_^-^, which were closely related to the hydrogeological conditions in varying geo-environmental zones, had significant impact on the distribution of groundwater microbes (Additional file 2: Fig. S14).

## Discussion

Ecological baselines are essential for reconciling arguments about maintenance of biological diversity, original state of biotic communities, and ecosystem functions [63]. The existence of ecological baseline on subsurface groundwater is still an important and open question due to the extreme susceptibility to pollution. The concept of a groundwater microbial ecological baseline (GMEB) is an extension of the ecological baseline of earth surface ecosystems [17, 18] and is proposed specifically for subsurface groundwater ecosystems where microbes are almost the only organisms present [64]. We define the GMEB as a reference for comparing microbial communities in groundwater affected by human intervention with those in the absence of human intervention. The GMEB has four unique characteristics: (1) the GMEB should be in pristine groundwater and need to be derived from “newly constructed wells” to avoid to the utmost extent the interference of human activities, (2) the GMEB should be capable of reflecting the whole bacterial community including uncultured bacterial species with help of the advanced high-throughput sequencing technology, (3) the GMEB should be determined based on a good number of samples taken from representative sites covering a typical variety of hydrological and geological environments at continental scale, and (4) the GMEB should be largely driven by deterministic processes in terms of specific niche. In the present work, we implemented a large-scale monitoring campaign to obtain first-hand data from “newly constructed wells” to establish the GMEB and parallel data from “reconstructed wells” to evaluate anthropogenic impacts on microbial community structures at the test sites. The stability of microbial communities in groundwater has been proved spatiotemporally with the proviso that habitats remained unchanged [65, 66]. The higher community similarity within the same geo-environment and its significant distance decay in pristine groundwater throughout China supported the fundamental assumption that similar biological components should be expected at congeneric environments in the absence of human intervention [20].

Recent progress in high-throughput sequencing has provided us with a relatively unbiased compositional snapshot of microbial communities [24] and helped us uncover the mysterious world of subsurface microbes. Based on the present unique bacterial dataset derived from pristine groundwater, we depicted the baseline patterns by comparing the microbial latitude diversity gradient in pristine groundwater at different burial depths and in the topsoil. Laterally, baseline microbes exhibited a unimodal LDG pattern with highest diversity at latitudes close to 40° N, suggesting mid-latitude of high humidity and warm temperature would provide optimum survival habitats for microbes. Vertically, the LDG got closer to those on the topsoil with decreasing burial depth [25, 58], indicating the divergent microbial pool at the surface would directly influence microbial diversity in shallower groundwater. In short, the geo-environment, as a complex macroscopic factor controlling hydrological connectivity and chemical characteristics of groundwater, has played an important role in shaping the biogeographic patterns of baseline microbes across China. Groundwater microbial diversity is highest in the Huanghuaihai-Yangtze River Delta Plain zone due to relatively frequent surface-groundwater interactions promoted by local hydrogeological characteristics including multi-fault structures, widespread loose and non-rock clay accumulation, and slow horizontal runoff [67].

Microbial ecological baseline patterns in pristine groundwater might be primarily mediated by certain dominant and key taxa [68]. *Proteobacteria*, the most typical habitat generalists [45], were confirmed as absolute dominators of groundwater microbial community. Driven by the mass propagation of their few taxa, *Proteobacteria* tended to have greater relative abundance in extreme environments, which would in turn inhibit local microbial diversity (Fig. S11, *r* = −0.54, *P* < 0.001). On the other hand, the majority of *Patescibacteria* members exhibited niche specialization and demonstrated significant declines in relative abundance and diversity with increasing well depth. *Patescibacteria* were characterized by small genome size, presence of potential attachment and adhesion proteins, and absence of numerous biosynthetic capacities, suggesting that they could not live alone and instead would be parasites or form mutualistic arrangements with other microorganisms [15, 16]. Network analysis further revealed the mediating role of *Patescibacteria* as keystone taxa in shallow phreatic water (Fig. 5b). Through anaerobic fermentative metabolism, certain members of *Patescibacteria* were capable of producing organic carbon, including hydrogen, acetate, formate, and ethanol, for other microbes [12, 14]. Moreover, *Patescibacteria* may promote and maintain the interconnectedness and connectivity of the microbial community via quorum sensing signals and potential co-metabolism [69]. Some phylotypes of *Patescibacteria* were unable to colonize successfully in absence of available symbiotic partners because of the scarcity of available niches, further accelerating decline in microbial diversity in deeper phreatic-water layer.

The existence of GMEB relies on niche differentiation with respect to microbes in pristine groundwater, implying the importance of deterministic processes in community assembly [34]. In surface water, microbial communities tend to be driven by stochastic processes due to strong flow-induced turbulence [70]. In pristine groundwater, however, microbial communities are predominantly shaped by deterministic processes due to restrictions of relatively isolated, stable, and high heterogeneous habitats, leading to the possible occurrence of a GMEB. The persistent march of selection by subterranean environmental constraints would preserve microorganisms capable of efficient energy utilization and/or special strategies of nutrient sequestration to better cope with low energy flux [6, 71]. Our study has indicated that a relatively high proportion of autotrophic microbes can exist in groundwater, being strongly influenced by specific electron acceptors or donors (e.g., HCO_3_^-^, Fe, Mn, and nitrate) (Additional file 2: Fig. S15). These findings could partially explain how microbial communities adapt to subterranean dark, anoxic, and nutrient-limited environments. From the perspective of assessing anthropogenic impact on groundwater ecosystems, shallower phreatic water should be of much greater significance for the establishment of GMEB considering its easier susceptibility to human footprints. Interestingly, environmental selection has been found to provide a relatively poor explanation of microbial community variation in deeper phreatic and confined groundwater, but this does not affect the claim about existence of a microbial baseline in shallower phreatic water (Additional file 2: Fig. S16). Beyond the scope of shallower phreatic water, a higher mean niche breadth of taxa has been observed due to increased proportions of habitat generalists with high biological adaptability through a long-term series of ecological successions [45], ultimately leading to relatively low diversity and high community homogeneity in deeper groundwater.

**Fig. 6:**
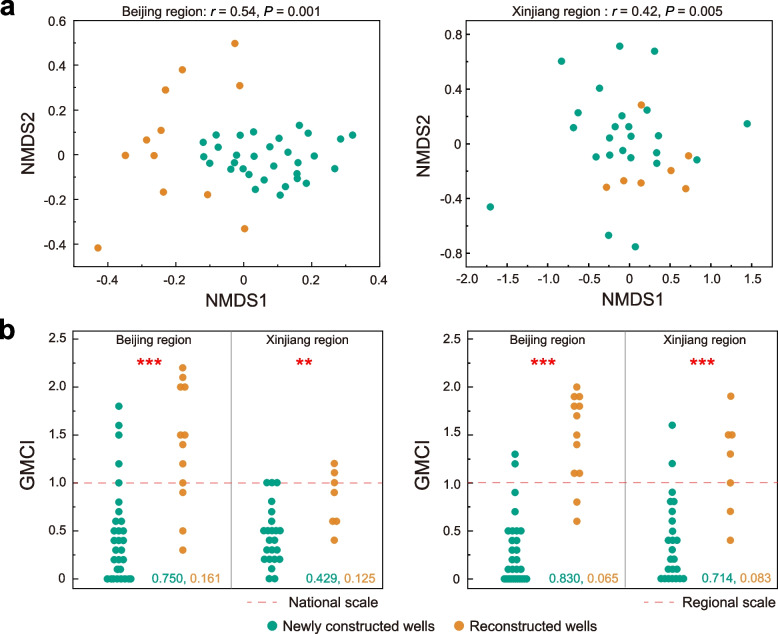
Evaluation of anthropogenic interferences on groundwater bacterial communities. **a** Non-metric multidimensional scaling (NMDS) analysis based on Bray–Curtis similarity showing compositional discrepancy on microbial community between newly constructed and reconstructed wells. Beijing and Xinjiang regions are selected as the representative regions suffering strong and weak human intervention, respectively. **b** Comparisons of GMCI assessment results of microbial communities in newly constructed and reconstructed wells. Left figure shows GMCI assessment results of two representative regions base on national baseline data, while right one is based on regional baseline data. The identified accurate rate (green) and error rate (yellow) are provided in the panel legend

Subterranean microbes are particularly sensitive to anthropogenic intervention in their evolutionary adaptations [72]. The GMEB suggests that similar microbial structures should be expected at congeneric environments in the absence of human intervention. Therefore, the anthropogenic impact on microbial community structures in the test sites could be evaluated by comparing with the baseline at reference sites with similar habitats [17, 18]. At a national scale, our monitoring results have indicated that anthropogenic perturbation did cause an increase in microbial diversity and alteration of community structure even at phylum level (Additional file 2: Fig. S17). To facilitate evaluation of anthropogenic impact in practical groundwater monitoring, we proposed Groundwater Microbial Community Index (GMCI), which integrated microbial diversity, key species, and biomarkers (see “Methods”). For GMCI≥1.0, the anthropogenic impact would be significant at specific test sites matched against the same reference group (Additional file 1: Table S9, S10, and S11), with larger GMCI indicating a stronger effect of human activity. To fully understand the effects of human activities on microbial ecological baselines in groundwater, we devised two categories of microbial baseline: one is the baseline at reference sites in regions experiencing intensive human intervention, such as the Beijing region, and the other is in regions with less human interference, such as the Xinjiang region. Without loss of generality, the difference in monitored community dissimilarity between newly constructed and reconstructed wells (Fig. 6a and Additional file 2: Fig. S18) in these two representative regions corresponded to the GMCI-based assessment results (Fig. 6b), which shed light on the potentials to establishing a feasible framework for human-impact evaluation under representative scenarios. It should be noted that the GMCI-based assessment had some obvious drawbacks. For example, the sequencing depth and sampling methods significantly influenced the resolution and accuracy of high-throughput sequencing, which required us to formulate standard monitoring methods for microbial communities. Noting the present inadequacy of GMCI data, priority should be given to the classification of reference groups and construction of a reference database for typical microbial habitats. At global scale, the establishment of GMEB system would be a new contribution to the decades-long International Geosphere-Biosphere Program (IGBP), which focused on possible anthropogenic influence and other factors that determine habitability of the earth [73].

## Conclusions

We confirmed the existence of the GMEB at continental scale by unveiling the biogeographic pattern of bacteria in pristine phreatic water based on a unique dataset derived from recent monitoring of 733 newly constructed wells in seven geo-environmental zones across China. The GMEB exhibits a latitudinal diversity gradient pattern which approximates that in topsoil with decreasing well depth, and the alpha diversity peaks in the belt around 40° N due to frequent groundwater-surface interactions facilitated by special geo-environments. We found that *Proteobacteria* was the dominator (contributing over half the total abundance) in groundwater, while *Patescibacteria* acted as hubs harmonizing symbiotic microbes in shallower phreatic aquifers and promoting the vertical decay of microbial communities downwards. We revealed the endogenous mechanism for microbial co-occurrence in shallower phreatic water, and the ideal exogenous conditions for baseline microbes predominantly driven by deterministic processes under varying geo-environments. Furthermore, we proposed GMCI-based assessment to facilitate evaluation of anthropogenic impact in practical groundwater monitoring, highlighting the fundamental importance of GMEB for health diagnosis and water security of underexplored groundwater ecosystems. In the long run, much more information is needed to enrich the reference database and continuously improve the system of reference groups constituted by microbes and their matched habitats. Multimetric approaches need to be developed that account for the combined effect of multiple attributes and provide an overall evaluation of the status of the microbial community under severe anthropogenic interference. In this regard, the concept of a “habitat ~ microbial reference ~ subterranean truth” system is recommended to reflect the relationship between geo-environment and microbial structure in groundwater ecosystems at regional, national, and global scales.

## Supplementary Information

**Additional file 1:** **Table S1.** Climatic characteristics, groundwater types, and environmental issues in different geo-environmental zones. **Table S2.** Sample sizes for each group, classified according to well type, burial depth, and geo-environmental zone. **Table S3.** Statistical description of QA/QC data for DNA extraction. **Table S4. **Detailed information on the grouping variables and statistical hypothesis for each of the analytical methods used in the study. **Table S5.** Polynomial regression of microbial taxonomic diversity with latitude in eastern (zone I, II, and III), middle (zone IV and V), and western (zone VI and VII) China for phreatic water of varying well depth ranges. **Table S6.** Linear and polynomial regression of relative abundance of dominant taxa (average relative abundance > 1%) at class, order, family, and genus level against well depth in phreatic water. **Table S7.** Key topological features of co-occurrence networks in phreatic water. **Table S8. **Normalized stochasticity ratio (NST) of microbial community assembly in groundwater based on taxonomic and phylogenetic beta diversity. **Table S9.** The weight and score of each indicator for GMIC based on baseline microbial data at a national scale. **Table S10.** The identified accurate rate and error rate of groundwater samples suffered by anthropogenic interferences for each indicator and GMIC based on baseline microbial data at anational scale. **Table S11.** The weight and score of each indicator for GMIC based on baseline microbial data at regional scale.

**Additional file 2:** **Fig. S1.** Taxonomic percentage of sequences (a) and OTUs (b) for the overall bacterial communities at phylum level. **Fig. S2.** a, Rank-abundance curves for bacterial community; b, Percentage numbers and relative abundance of dominant OTUs (average abundance > 0.01%) in groundwater sampled from different types of wells. **Fig. S3.** Longitudinal distributions of microbial taxonomic and phylogenetic diversity in groundwater. Red solid and black dashed lines show polynomial and linear fits based on ordinary least-square regression analysis, with the shaded area representing 95% confidence intervals. Adjusted *R*^2^ of the polynomial fits and Pearson’s *r* of the linear fits are provided. **Fig. S4.** Violin plot comparing microbial diversity across seven geo-environmental zones in phreatic water of varying burial depth ranges. **Fig. S5.** Distance-decay curves showing the relationship between geographic distance and community similarity. Red and blue lines denote the least-square linear regressions between geo-environmental zones and within the same geo-environmental zone. Slope and *P *values (one-sided) for regression slopes are stated. **Fig. S6.** Depth stratification of microbial communities in pristine groundwater. a, Alpha-diversity variation with well depth in newly constructed wells (*n* = 733), confined water (*n* = 229), and phreatic water (*n* = 504). Black and red lines show linearand polynomial regressions, with shaded representing 95% confidence intervals. b, Non-metric multidimensional scaling (NMDS) analysis based on Bray-Curtis similarity showing compositional variation with well depth in phreatic water. The color gradient denotes the well depth of each sample. c, Boxplot of community similarity in phreatic water for three well depth ranges. Asterisks denote the significance of correlations (***P <0.001). **Fig. S7.** Vertical distributions of microbial diversity inphreatic water under varying geo-environments. Adjusted *R*^2^ of the polynomial fits are provided. **Fig. S8.** a, Distance-decay relationships (DDRs) showing community similarity against geographic distance between sampling sites in phreatic water of three well depth ranges. Red lines denote ordinary least-squares linear regressions, with the shaded area representing 95% confidence intervals. Slope of DDRs, mantel Spearman correlations (*r*), and probabilities (*P*) are listed inthe legends. b, Variance partition analysis showing relative contributions of environmental (Env.) and geographical (Geo.) factors and their combined effect on community variations based on Bray-Curtis similarity. **Fig. S9.** LEfSe cladogram of microbial community obtained for varying well-depth ranges (a), geo-environmental zones at shallower (b), medium (c) and deeper (d) phreatic water. All detected taxa with average relative abundance ≥ 0.5% were assigned to domain (innermost), phylum,class, order, family, and genus (outermost). Differentially abundant taxa (biomarkers) are colored according to their most abundant regions. **Fig. S10.** Variation in relative abundance of dominant phylawith well depth in phreatic water. Red and blue lines show linear and polynomial fits based on ordinary least-square regression, with the shaded areas representing 95% confidence intervals. Adjusted *R*^2^ of the polynomial fitsand Pearson’s *r* of the linear fits are provided. Variation in relative abundance of dominant phyla with well depth in phreatic water. **Fig. S11.** Composition and distribution of *Patescibacteria* in groundwater. a, Taxonomic tree of identified *Patescibacteria* OTUs in groundwater. The color of circles indicates taxonomic information of each OTU, and the size of circles is proportional to the relative abundance. The grey circles represent the OTUs with no affiliation at the corresponding taxonomic level. b, Average relative abundance of *Patescibacteria* classes in varying types of wells and geo-environmental zones. c, Variation of the relative abundance of *Patescibacteria* classes with well depth in phreatic water. The blue lines show the polynomial fit based on ordinary least squares regression, with the shaded areas representing 95% confidence intervals. *0.01 < *P* < 0.05, **0.001 < *P* < 0.01 and ***P < 0.001. **Fig. S12.** Relationship between microbial diversity and relative abundance of *Proteobacteria*, *Parcubacteria*, *Chloroflexi*, and *Verrucomicrobiota* in reconstructed wells (a), phreatic water (b), and confined water (c). Red lines indicate ordinary least square linear regressions across all samples in each habitat. Shaded areas represent 95% confidence intervals. Adjusted Pearson correlations (*r*) of the linear fits are provided. **Fig. S13.** Ecological drivers of microbial community in phreatic and confined water. Pairwise Spearman correlation coefficients between environmental parameters are indicated by the color gradient. Asterisks denote the significance of correlations (*0.01 < *P*< 0.05, **0.001 < *P* < 0.01, and***P < 0.001). Taxonomic composition (based on Bray-Curtis distance) was related to each environmental parameter (basedon Euclidean distance) by Mantel tests. Edge width corresponds to Mantel *r* statistics, and edge color denotes statistical significance. **Fig. S14.** a, Constrained correspondence analyses (CCA) reveal environmental parameters governing the distribution of microbial communities in phreatic and confined water. b, The panels show respective geochemical signatures as percentagesof chloride (Cl^−^), sulfate (SO_4_^2−^), and thesum of carbonate (CO_3_^2−^) and bicarbonate (HCO_3_^−^) ions in phreatic and confined water for the seven geo-environments in China. **Fig. S15.** Cluster heatmap analysis demonstrating that microbial dominant taxa (average relative abundance > 1%) at phylum (a) and genus (b) level display abundance patterns corresponding to geochemical parameters. **Fig. S16.** Heatmap showing Pearson correlation between main geographical and environmental factors and the diversity and structure of microbial communities in phreatic water for different well depth groups. Asterisks denote the significance of correlations (*0.01< *P* < 0.05, **0.001 < *P* < 0.01, and ***P < 0.001). **Fig. S17.** Comparison of diversity and composition of microbial communities between reconstructed and newly constructed wells. a, Boxplots showing that both taxonomic and phylogenetic diversities in reconstructed wells are significantly higher thanin newly constructed wells. The hinges show the 25th, 50th, and 75th percentiles. b, Sankey diagrams showing the relative abundances of dominant phyla (> 1%) in reconstructed and newly constructedwells. **Fig. S18.** a, LEfSe analysis identifying the groundwater biomarker of reconstructed (blue) and newly constructed wells (red). All detected taxa (relative abundance ≥ 0.5%) are assigned to domain (innermost), phylum, class, order, family, and genus (outermost). Taxa meeting an LDA significant threshold of > 4.0 are selected as most likely to explain differences in microbial communities between reconstructed and newly constructed wells. b, Comparison of average relative abundance of biomarkers between reconstructed and newly constructed wells in Beijing and Xinjiang regions.

## Data Availability

All the raw datasets supporting the findings of this article are available in the NCBI Sequence Read Archive under BioProject number PRJNA692269.

## References

[1] de Graaf IEM, Gleeson T, van Beek LPH, Sutanudjaja EH, Bierkens MFP (2019). Environmental flow limits to global groundwater pumping. Nature.

[2] Griebler C, Lueders T (2009). Microbial biodiversity in groundwater ecosystems. Freshw Biol.

[3] McDonough LK, Santos IR, Andersen MS, O'Carroll DM, Rutlidge H, Meredith K (2020). Changes in global groundwater organic carbon driven by climate change and urbanization. Nat Commun.

[4] Whitman WB, Coleman DC, Wiebe WJ (1998). Prokaryotes: the unseen majority. Proc Natl Acad Sci USA.

[5] Magnabosco C, Lin LH, Dong H, Bomberg M, Ghiorse W, Stan-Lotter H (2018). The biomass and biodiversity of the continental subsurface. Nat Geosci.

[6] Probst AJ, Ladd B, Jarett JK, Geller-McGrath DE, Sieber CMK, Emerson JB (2018). Differential depth distribution of microbial function and putative symbionts through sediment- hosted aquifers in the deep terrestrial subsurface. Nat Microbiol.

[7] Wang S, Zhu G, Zhuang L, Li Y, Liu L, Lavik G (2019). Anaerobic ammonium oxidation is a major N-sink in aquifer systems around the world. ISME J.

[8] Chiriac CM, Baricz A, Szekeres E, Rudi K, Dragos N, Coman C (2018). Microbial composition and diversity patterns in deep hyperthermal aquifers from the western plain of Romania. Microb Ecol.

[9] Hubalek V, Wu X, Eiler A, Buck M, Heim C, Dopson M (2016). Connectivity to the surface determines diversity patterns in subsurface aquifers of the Fennoscandian shield. ISME J.

[10] Seyler LM, Trembath-Reichert E, Tully BJ, Huber JA (2021). Time-series transcriptomics from cold, oxic subseafloor crustal fluids reveals a motile, mixotrophic microbial community. ISME J.

[11] Brown CT, Hug LA, Thomas BC, Sharon I, Castelle CJ, Singh A (2015). Unusual biology across a group comprising more than 15% of domain Bacteria. Nature.

[12] Anantharaman K, Brown CT, Hug LA, Sharon I, Castelle CJ, Probst AJ (2016). Thousands of microbial genomes shed light on interconnected biogeochemical processes in an aquifer system. Nat Commun.

[13] Hug LA, Baker BJ, Anantharaman K, Brown CT, Probst AJ, Castelle CJ (2016). A new view of the tree of life. Nat Microbiol.

[14] He C, Keren R, Whittaker ML, Farag IF, Doudna JA, Cate JHD (2021). Genome-resolved metagenomics reveals site-specific diversity of episymbiotic CPR bacteria and DPANN archaea in groundwater ecosystems. Nat Microbiol.

[15] Lemos LN, Medeiros JD, Dini-Andreote F, Fernandes GR, Varani AM, Oliveira G (2020). Genomic signatures and co-occurrence patterns of the ultra-small Saccharimonadia (phylum CPR/Patescibacteria) suggest a symbiotic lifestyle (vol 28, pg 4259, 2019). Mol Ecol.

[16] Tian R, Ning D, He Z, Zhang P, Spencer SJ, Gao S (2020). Small and mighty: adaptation of superphylum Patescibacteria to groundwater environment drives their genome simplicity. Microbiome.

[17] Burger J, Gochfeld M, Powers CW, Greenberg M (2007). Defining an ecological baseline for restoration and natural resource damage assessment of contaminated sites: the case of the department of energy. J Environ Plan Manag.

[18] Linder HL, Horne JK, Ward EJ (2017). Modeling baseline conditions of ecological indicators: marine renewable energy environmental monitoring. Ecol Indic.

[19] Hobday AJ (2011). Sliding baselines and shuffling species: implications of climate change for marine conservation. Mar Ecol Evol Persp.

[20] Lei L, Sun JS, Borthwick AGL, Fang Y, Ma JP, Ni JR (2013). Dynamic evaluation of intertidal wetland sediment quality in a bay system. J Environ Inform.

[21] Griebler C, Avramov M (2015). Groundwater ecosystem services: a review. Freshw Sci.

[22] Khanoranga Khalid S (2019). An assessment of groundwater quality for irrigation and drinking purposes around brick kilns in three districts of Balochistan province, Pakistan, through water quality index and multivariate statistical approaches. J Geochem Explor.

[23] Meyer KM, Memiaghe H, Korte L, Kenfack D, Alonso A, Bohannan BJM (2018). Why do microbes exhibit weak biogeographic patterns?. ISME J.

[24] Reuter JA, Spacek DV, Snyder MP (2015). High-throughput sequencing technologies. Mol Cell.

[25] Bahram M, Hildebrand F, Forslund SK, Anderson JL, Soudzilovskaia NA, Bodegom PM (2018). Structure and function of the global topsoil microbiome. Nature.

[26] Sunagawa S, Coelho LP, Chaffron S, Kultima JR, Labadie K, Salazar G (2015). Structure and function of the global ocean microbiome. Science.

[27] Carlson HK, Price MN, Callaghan M, Aaring A, Chakraborty R, Liu H (2019). The selective pressures on the microbial community in a metal-contaminated aquifer. ISME J.

[28] Wang L, Yin Z, Jing C (2020). Metagenomic insights into microbial arsenic metabolism in shallow groundwater of Datong basin China. Chemosphere.

[29] Mikucki JA, Auken E, Tulaczyk S, Virginia RA, Schamper C, Sorensen KI (2015). Deep groundwater and potential subsurface habitats beneath an Antarctic dry valley. Nat Commun.

[30] Power JF, Carere CR, Lee CK, Wakerley GLJ, Evans DW, Button M (2018). Microbial biogeography of 925 geothermal springs in New Zealand. Nat Commun.

[31] Liu S, Wang H, Chen L, Wang J, Zheng M, Liu S (2020). Comammox nitrospira within the Yangtze River continuum: community, biogeography, and ecological drivers. ISME J.

[32] Archer SDJ, Lee KC, Caruso T, Maki T, Lee CK, Carys SC (2019). Airborne microbial transport limitation to isolated Antarctic soil habitats. Nat Microbiol.

[33] Fodelianakis S, Moustakas A, Papageorgiou N, Manoli O, Tsikopoulou I, Michoud G (2017). Modified niche optima and breadths explain the historical contingency of bacterial community responses to eutrophication in coastal sediments. Mol Ecol.

[34] Pernthaler J (2017). Competition and niche separation of pelagic bacteria in freshwater habitats. Environ Microbiol.

[35] Welch JLM, Ramirez-Puebla ST, Borisy GG (2020). Oral microbiome geography: micron-scale habitat and niche. Cell Host Microbe.

[36] Caporaso JG, Lauber CL, Walters WA, Berg-Lyons D, Lozupone CA, Turnbaugh PJ (2011). Global patterns of 16S rRNA diversity at a depth of millions of sequences per sample. Proc Natl Acad Sci USA.

[37] Caporaso JG, Kuczynski J, Stombaugh J, Bittinger K, Bushman FD, Costello EK (2010). QIIME allows analysis of high-throughput community sequencing data. Nat Methods.

[38] Edgar RC (2013). UPARSE: highly accurate OTU sequences from microbial amplicon reads. Nat Methods.

[39] Wang Q, Garrity GM, Tiedje JM, Cole JR (2007). Naive Bayesian classifier for rapid assignment of rRNA sequences into the new bacterial taxonomy. Appl Environ Microbiol.

[40] Delgado-Baquerizo M, Oliverio AM, Brewer TE, Benavent-Gonzalez A, Eldridge DJ, Bardgett RD (2018). A global atlas of the dominant bacteria found in soil. Science.

[41] Kumar S, Stecher G, Li M, Knyaz C, Tamura K (2018). MEGA X: molecular evolutionary genetics analysis across computing platforms. Mol Biol Evol.

[42] Schloss PD, Westcott SL, Ryabin T, Hall JR, Hartmann M, Hollister EB (2009). Introducing mothur: open-source, platform-independent, community-supported software for describing and comparing microbial communities. Appl Environ Microbiol.

[43] Jiao S, Yang Y, Xu Y, Zhang J, Lu Y (2020). Balance between community assembly processes mediates species coexistence in agricultural soil microbiomes across eastern China. ISME J.

[44] Banerjee S, Schlaeppi K, van der Heijden MGA (2018). Keystone taxa as drivers of microbiome structure and functioning. Nat Rev Microbiol.

[45] Logares R, Lindstrom ES, Langenheder S, Logue JB, Paterson H, Laybourn-Parry J (2013). Biogeography of bacterial communities exposed to progressive long-term environmental change. ISME J.

[46] Wu W, Lu H-P, Sastri A, Yeh Y-C, Gong G-C, Chou W-C (2018). Contrasting the relative importance of species sorting and dispersal limitation in shaping marine bacterial versus protist communities. ISME J.

[47] Jabot F, Etienne RS, Chave J (2008). Reconciling neutral community models and environmental filtering: theory and an empirical test. Oikos.

[48] Feinstein LM, Blackwood CB (2012). Taxa-area relationship and neutral dynamics influence the diversity of fungal communities on senesced tree leaves. Environ Microbiol.

[49] Guo X, Feng J, Shi Z, Zhou X, Yuan M, Tao X (2018). Climate warming leads to divergent succession of grassland microbial communities. Nat Clim Chang.

[50] Ning D, Deng Y, Tiedje JM, Zhou J (2019). A general framework for quantitatively assessing ecological stochasticity. Proc Natl Acad Sci USA.

[51] Grace JB, Bollen KA (2008). Representing general theoretical concepts in structural equation models: the role of composite variables. Environ Ecol Stat.

[52] Roy AH, Rosemond AD, Paul MJ, Leigh DS, Wallace JB (2003). Stream macroinvertebrate response to catchment urbanisation (Georgia, USA). Freshw Biol.

[53] Wu L, Ning D, Zhang B, Li Y, Zhang P, Shan X (2019). Global diversity and biogeography of bacterial communities in wastewater treatment plants. Nat Microbiol.

[54] Liu L, Yang J, Yu Z, Wilkinson DM (2015). The biogeography of abundant and rare bacterioplankton in the lakes and reservoirs of China. ISME J.

[55] Vasquez-Ponce F, Higuera-Llanten S, Pavlov MS, Marshall SH, Olivares-Pacheco J (2018). Phylogenetic MLSA and phenotypic analysis identification of three probable novel Pseudomonas species isolated on King George Island, South Shetland. Antarctica Braz J Microbiol.

[56] Gleeson T, Befus KM, Jasechko S, Luijendijk E, Cardenas MB (2016). The global volume and distribution of modern groundwater. Nat Geosci.

[57] Clark DR, Underwood GJC, McGenity TJ, Dumbrell AJ (2021). What drives study-dependent differences in distance-decay relationships of microbial communities?. Glob Ecol Biogeogr.

[58] Zhang X, Liu S, Wang J, Huang Y, Freedman Z, Fu S (2020). Local community assembly mechanisms shape soil bacterial beta diversity patterns along a latitudinal gradient. Nat Commun.

[59] Herault B (2007). Reconciling niche and neutrality through the Emergent Group approach. Plant Ecol Evol Syst.

[60] Chen J, Wang P, Wang C, Wang X, Miao L, Liu S (2020). Fungal community demonstrates stronger dispersal limitation and less network connectivity than bacterial community in sediments along a large river. Environ Microbiol.

[61] Zhou L, Li H, Zhang Y, Han S, Xu H (2016). Sphingomonas from petroleum-contaminated soils in Shenfu, China and their PAHs degradation abilities. Braz J Microbiol.

[62] Mendes LW, Kuramae EE, Navarrete AA, van Veen JA, Tsai SM (2014). Taxonomical and functional microbial community selection in soybean rhizosphere. ISME J.

[63] Arcese P, Sinclair ARE (1997). The role of protected areas as ecological baselines. J Wildl Manage.

[64] Danielopol DL, Griebler C (2008). Changing paradigms in groundwater ecology - from the 'living fossils' tradition to the 'new groundwater ecology'. Int Rev Hydrobiol.

[65] Sirisena KA, Daughney CJ, Moreau M, Ryan KG, Chambers GK (2014). Relationships between molecular bacterial diversity and chemistry of groundwater in the Wairarapa Valley, New Zealand. N Z J Mar Freshw Res.

[66] Farnleitner AH, Wilhartitz I, Ryzinska G, Kirschner AKT, Stadler H, Burtscher MM (2005). Bacterial dynamics in spring water of alpine karst aquifers indicates the presence of stable autochthonous microbial endokarst communities. Environ Microbiol.

[67] Wang Y, Zhou S (2009). Ar-40/Ar-39 dating constraints on the high-angle normal faulting along the southern segment of the Tan-Lu fault system: an implication for the onset of eastern China rift-systems. J Asian Earth Sci.

[68] Zhalnina K, Louie KB, Hao Z, Mansoori N, da Rocha UN, Shi S (2018). Dynamic root exudate chemistry and microbial substrate preferences drive patterns in rhizosphere microbial community assembly. Nat Microbiol.

[69] Bernard C, Lannes R, Li Y, Bapteste E, Lopez P (2020). Rich repertoire of quorum sensing protein coding sequences in CPR and DPANN associated with interspecies and interkingdom communication. Msystems.

[70] Liu T, Zhang AN, Wang J, Liu S, Jiang X, Dang C (2018). Integrated biogeography of planktonic and sedimentary bacterial communities in the Yangtze River. Microbiome.

[71] Hoehler TM, Jorgensen BB (2013). Microbial life under extreme energy limitation. Nat Rev Microbiol.

[72] Castano-Sanchez A, Hose GC, Reboleira ASPS (2020). Ecotoxicological effects of anthropogenic stressors in subterranean organisms: a review. Chemosphere.

[73] Abelson PH (1986). The international geospher-biospher progam. Science.

