# Supplementary material for: May microbial ecological baseline exist in continental groundwater?
Source: Microbiome. 2023 Jul 19;11:152. doi: 10.1186/s40168-023-01572-4 (PMC10355068; doi:10.1186/s40168-023-01572-4)
Supplement: Supplementary file 2 — Additional file 1: Table S1. Climatic characteristics, groundwater types, and environmental issues in different geo-environmental zones. Table S2. Sample sizes for each group, classified according to well type, burial depth, and geo-environmental zone. Table S3. Statistical description of QA/QC data for DNA extraction. Table S4. Detailed information on the grouping variables and statistical hypothesis for each of the analytical methods used in the study. Table S5. Polynomial regression of microbial taxonomic diversity with latitude in eastern (zone I, II, and III), middle (zone IV and V), and western (zone VI and VII) China for phreatic water of varying well depth ranges. Table S6. Linear and polynomial regression of relative abundance of dominant taxa (average relative abundance > 1%) at class, order, family, and genus level against well depth in phreatic water. Table S7. Key topological features of co-occurrence networks in phreatic water. Table S8. Normalized stochasticity ratio (NST) of microbial community assembly in groundwater based on taxonomic and phylogenetic beta diversity. Table S9. The weight and score of each indicator for GMIC based on baseline microbial data at a national scale. Table S10. The identified accurate rate and error rate of groundwater samples suffered by anthropogenic interferences for each indicator and GMIC based on baseline microbial data at anational scale. Table S11. The weight and score of each indicator for GMIC based on baseline microbial data at regional scale. [file 40168_2023_1572_MOESM1_ESM.docx]

**Additional file 1**

**May microbial ecological baseline exist in continental groundwater?**

**Authors**: Sining Zhong^1,2,3^, Shungui Zhou^3^, Shufeng Liu^1^, Jiawen Wang^1^, Chenyuan Dang^1^, Qian Chen^1,4^, Jinyun Hu^1^, Shanqing Yang^1^, Chunfang Deng^1^, Wenpeng Li^5^, Juan Liu^1^, Alistair G.L. Borthwick^6, 7^, Jinren Ni^1,2^*

**Author affiliations**:

^1^College of Environmental Sciences and Engineering, Peking University; Key Laboratory of Water and Sediment Sciences, Ministry of Education, Beijing 100871, P. R. China

^2^State Environmental Protection Key Laboratory of All Material Fluxes in River Ecosystems, Beijing 100871, P. R. China

^3^Fujian Agriculture and Forestry University, College of Resources and Environment, Fujian Provincial Key Laboratory of Soil Environment Health and Regulation, Fuzhou 350002, P. R. China

^4^State Key Laboratory of Plateau Ecology and Agriculture, Qinghai University, Xining 810016, P. R. China

^5^Center for Groundwater Monitoring, China Institute of Geo-environmental Monitoring, Beijing 100081, P. R. China

^6^School of Engineering, Computing and Mathematics, University of Plymouth, Drake Circus, Plymouth PL8 4AA, UK.

***Corresponding author:** Jinren Ni

Postal address: Peking University, No. 5 Yiheyuan Road, Beijing 100871, P. R. China

Telephone number: +86-10-62751185

E-mail address: jinrenni@pku.edu.cn

Table S1 Climatic characteristics, groundwater types, and environmental issues in different geo-environmental zones.

| Zone | Climate characteristic^1^ | Groundwater type^2^ | Groundwater environmental issue^1^ |
| --- | --- | --- | --- |
| Ⅰ Northeast Plain-Mountain zone | Temperate monsoon climate | Basement fissure water and sand- gravel bed pore water | Mining pollution, chemical industrial pollution |
| Ⅱ Huanghuaihai-Yangtze River Delta Plain zone | Temperate semi-humid climate | Sand- gravel bed pore water | Overexploitation, salinization |
| Ⅲ South China Bedrock Foothill zone | Subtropical humid and monsoon climate | Basement fissure water | - |
| Ⅳ Northwest Loess Plateau zone | Temperate semi-humid and semi-arid climate | Loess pore water | Primary inferior groundwater, mining pollution |
| Ⅴ Southwest China Karst Rock-Mountain zone | Subtropical humid and monsoon climate | Karst fissure cave water | Surface pollution invasion |
| Ⅵ Northwest Arid Desert zone | Temperate continental climate | Basement fissure water and desert pore water | Overexploitation for agricultural irrigation |
| Ⅶ Qinghai-Tibet Plateau Alpine Frozen Soil zone | Plateau and mountain climate | Pore-fissure water in plateau frozen earth | Solute increase due to permafrost degradation |

Data sources: 1. China Geological Survey (<https://www.cgs.gov.cn/>); 2. Hydrogeologic Map of China (China Cartographic Publishing House, 1994)

Table S2. Sample sizes for each group, classified according to well type, burial depth, and geo-environmental zone.

| Zone | Phreatic water | 0-20 m | 0-40 m | 40-80 m | > 80 m | Confined water | Reconstructed well |
| --- | --- | --- | --- | --- | --- | --- | --- |
| Total | 504 | 70 | 216 | 182 | 107 | 229 | 130 |
| Ⅰ | 94 | 11 | 53 | 34 | 7 | 20 | 23 |
| Ⅱ | 162 | 22 | 75 | 71 | 16 | 99 | 57 |
| Ⅲ | 47 | 11 | 20 | 9 | 18 | 40 | 1 |
| Ⅳ | 77 | 15 | 30 | 27 | 20 | 33 | 25 |
| Ⅴ | 48 | 1 | 6 | 12 | 30 | 16 | 2 |
| Ⅵ | 34 | 2 | 9 | 14 | 11 | 19 | 17 |
| Ⅶ | 42 | 9 | 22 | 15 | 5 | 2 | 5 |

Table S3. Statistical description of QA/QC data for DNA extraction.

|  | Concentration (ng/µl) | OD260/280 | OD260/230 |
| --- | --- | --- | --- |
| Sample size | 863 | 863 | 863 |
| Method | NanoDrop2000 | NanoDrop2000 | NanoDrop2000 |
| Average | 45.3 | 1.81 | 0.86 |
| Standard error | 22.6 | 0.08 | 0.23 |
| Minimum value | 9.1 | 1.79 | 0.35 |
| Maximum value | 252.8 | 2.05 | 1.62 |

Table S4 Detailed information on the grouping variables and statistical hypothesis for each of the analytical methods used in the study.

| Statistical method | Variable | Group | Statistical hypothesis |
| --- | --- | --- | --- |
| Kruskal-Wallis test | Bacterial taxa | Well-depth ranges and geo-environmental zones | All groups drawn from populations with the same median |
| Wilcox test | Core microbial taxa | Well type (newly constructed and reconstructed wells) | All groups drawn from populations with the same median |
| ANOVA | Community diversity and structure | Well-depth ranges and geo-environmental zones | No significant difference in average values of all the groups |
| ANOSIM | Community diversity and structure | Well-depth ranges and geo-environmental zones | All groups drawn from the same population |

Table S5 Polynomial regression of microbial taxonomic diversity with latitude in eastern (zone Ⅰ, Ⅱ, and Ⅲ), middle (zone Ⅳ and Ⅴ), and western (zone Ⅵ and Ⅶ) China for phreatic water of varying well depth ranges.

| Well depth range | Eastern China | | Middle China | | Western China | |
| --- | --- | --- | --- | --- | --- | --- |
|  | *R*^2^ | *P* -value | *R*^2^ | *P* -value | *R*^2^ | *P* -value |
| 0-20 m | 0.15 | 0.038* | 0.26 | 0.191 | 0.03 | 0.688 |
| 20-40 m | 0.12 | 0.002** | 0.01 | 0.808 |  |  |
| 40-80 m | 0.01 | 0.830 | 0.16 | 0.048* | 0.06 | 0.464 |
| > 80 m | 0.05 | 0.25 | 0.02 | 0.587 | 0.05 | 0.710 |

Table S6 Linear and polynomial regression of relative abundance of dominant taxa (average relative abundance > 1%) at class, order, family, and genus level against well depth in phreatic water.

| Taxonomic level | Name | Abundance (%) | Linear fit | |  | | Polynomial fit | |
| --- | --- | --- | --- | --- | --- | --- | --- | --- |
|  |  |  | *R*^2^ | *P* -value | | *R*^2^ | | *P* -value |
| Class | *Gammaproteobacteria* | 41.44 | 0.04 | <0.001*** | | 0.04 | | <0.001*** |
|  | *Alphaproteobacteria* | 9.95 | 0.02 | <0.001*** | | 0.02 | | <0.001*** |
|  | *Bacteroidia* | 7.76 | 0.00 | 0.652 | | 0.00 | | 0.694 |
|  | *Campylobacteria* | 6.19 | 0.00 | 0.234 | | 0.00 | | 0.451 |
|  | *Actinobacteria* | 4.20 | 0.00 | 0.776 | | 0.00 | | 0.358 |
|  | *Bacilli* | 2.43 | 0.00 | 0.699 | | 0.00 | | 0.903 |
|  | *Clostridia* | 1.88 | 0.00 | 0.296 | | 0.00 | | 0.360 |
|  | *Parcubacteria* | 1.79 | 0.03 | <0.001*** | | 0.05 | | <0.001*** |
|  | *Methylomirabilia* | 1.70 | 0.02 | <0.001*** | | 0.03 | | <0.001*** |
|  | *Saccharimonadia* | 1.33 | 0.00 | 0.516 | | 0.00 | | 0.255 |
|  | *Thermodesulfovibrionia* | 1.13 | 0.01 | 0.015* | | 0.01 | | 0.028* |
| Order | *Burkholderiales* | 25.67 | 0.01 | 0.013* | | 0.01 | | 0.047* |
|  | *Pseudomonadales* | 10.01 | 0.01 | 0.008** | | 0.01 | | 0.027* |
|  | *Campylobacterales* | 6.19 | 0.00 | 0.234 | | 0.00 | | 0.451 |
|  | *Sphingomonadales* | 4.79 | 0.02 | 0.002** | | 0.02 | | 0.004** |
|  | *Flavobacteriales* | 4.06 | 0.00 | 0.624 | | 0.00 | | 0.562 |
|  | *Micrococcales* | 2.44 | 0.00 | 0.269 | | 0.00 | | 0.511 |
|  | *Rhizobiales* | 2.03 | 0.00 | 0.378 | | 0.00 | | 0.156 |
|  | *Bacteroidales* | 1.56 | 0.00 | 0.684 | | 0.00 | | 0.908 |
|  | *Saccharimonadales* | 1.33 | 0.00 | 0.516 | | 0.00 | | 0.255 |
|  | *Caulobacterales* | 1.30 | 0.00 | 0.166 | | 0.00 | | 0.341 |
|  | *Aeromonadales* | 1.12 | 0.00 | 0.557 | | 0.01 | | 0.017* |
| Family | *Comamonadaceae* | 13.25 | 0.04 | <0.001*** | | 0.05 | | <0.001*** |
|  | *Pseudomonadaceae* | 6.54 | 0.00 | 0.254 | | 0.00 | | 0.398 |
|  | *Sphingomonadaceae* | 4.79 | 0.02 | 0.002** | | 0.02 | | 0.004** |
|  | *Sulfurimonadaceae* | 4.72 | 0.00 | 0.089 | | 0.00 | | 0.202 |
|  | *Flavobacteriaceae* | 3.67 | 0.00 | 0.666 | | 0.00 | | 0.636 |
|  | *Moraxellaceae* | 3.46 | 0.03 | <0.001*** | | 0.03 | | <0.001*** |
|  | *Rhodocyclaceae* | 3.01 | 0.00 | 0.328 | | 0.01 | | 0.045* |
|  | *Gallionellaceae* | 2.39 | 0.00 | 0.196 | | 0.01 | | 0.074 |
|  | *Oxalobacteraceae* | 2.22 | 0.00 | 0.582 | | 0.00 | | 0.851 |
|  | *Micrococcaceae* | 1.88 | 0.00 | 0.195 | | 0.00 | | 0.431 |
|  | *Burkholderiaceae* | 1.37 | 0.00 | 0.858 | | 0.00 | | 0.699 |
|  | *Caulobacteraceae* | 1.28 | 0.00 | 0.150 | | 0.00 | | 0.314 |
|  | *Arcobacteraceae* | 1.10 | 0.00 | 0.371 | | 0.00 | | 0.669 |
|  | *Aeromonadaceae* | 1.09 | 0.00 | 0.548 | | 0.01 | | 0.017* |

Table S6 (Continued).

| Taxonomic level | Name | Abundance  (%) | Linear fit | |  | | Polynomial fit | |
| --- | --- | --- | --- | --- | --- | --- | --- | --- |
|  |  |  | *R*^2^ | *P* -value | | *R*^2^ | | *P* -value |
| Genus | *Pseudomonas* | 6.54 | 0.00 | 0.254 | | 0.00 | | 0.398 |
|  | *Sulfuricurvum* | 3.48 | 0.01 | 0.053 | | 0.00 | | 0.131 |
|  | *Flavobacterium* | 3.36 | 0.00 | 0.620 | | 0.00 | | 0.407 |
|  | *Aquabacterium* | 3.35 | 0.00 | 0.091 | | 0.00 | | 0.237 |
|  | *Hydrogenophaga* | 3.17 | 0.04 | <0.001*** | | 0.04 | | <0.001*** |
|  | *Novosphingobium* | 2.67 | 0.01 | 0.038* | | 0.00 | | 0.118 |
|  | *Acinetobacter* | 2.05 | 0.02 | <0.001*** | | 0.02 | | 0.004** |
|  | *Rhodoferax* | 1.89 | 0.00 | 0.544 | | 0.00 | | 0.409 |
|  | *Gallionella* | 1.57 | 0.01 | 0.056 | | 0.00 | | 0.144 |
|  | *Acidovorax* | 1.32 | 0.01 | 0.022* | | 0.01 | | 0.055 |
|  | *Sulfurimonas* | 1.23 | 0.00 | 0.977 | | 0.00 | | 0.991 |

Table S7 Key topological features of co-occurrence networks in phreatic water.

| Topological feature | Total | 0-20 m | 20-40 m | 40-60 m | 60-80 m | > 80 m |
| --- | --- | --- | --- | --- | --- | --- |
| Number of nodes | 398 | 475 | 467 | 470 | 359 | 401 |
| Number of edges | 1923 | 2230 | 1924 | 2397 | 2007 | 1909 |
| Average degree | 9.66 | 9.39 | 8.24 | 10.20 | 11.20 | 9.72 |
| Positive connectivity (%) | 100 | 100 | 100 | 100 | 100 | 93.98 |
| Negative connectivity (%) | 0 | 0 | 0 | 0 | 0 | 6.02 |
| Clustering coefﬁcient | 0.60 | 0.47 | 0.53 | 0.50 | 0.54 | 0.48 |
| Graph density | 0.024 | 0.02 | 0.018 | 0.022 | 0.031 | 0.026 |
| Average path length | 5.43 | 4.79 | 5.78 | 5.38 | 4.86 | 3.90 |
| Modularity | 0.62 | 0.60 | 0.73 | 0.65 | 0.48 | 0.76 |

Table S8 Normalized stochasticity ratio (NST) of microbial community assembly in groundwater based on taxonomic and phylogenetic beta diversity.

| Habitats | Taxonomic beta diversity | Phylogenetic beta diversity |
| --- | --- | --- |
| Reconstructed wells | 20.66% | 24.21% |
| Newly constructed wells | 31.70% | 32.52% |
| Phreatic water (Total) | 27.99% | 30.14% |
| 0-40 m (Phreatic water) | 25.65% | 27.91% |
| 40-80 m (Phreatic water) | 26.92% | 27.33% |
| > 80 m (Phreatic water) | 30.44% | 33.56% |
| Confined water | 43.98% | 47.67% |

Table S9 The weight and score of each indicator for GMIC based on baseline microbial data at a national scale.

| Indicator | Weight | Score | | | |
| --- | --- | --- | --- | --- | --- |
|  |  | 3 | 2 | 1 | 0 |
| Phylogenetic diversity | 0.1 | O/E > 2.0 | 1.5-2.0 | 1.2-1.5 | < 1.2 |
| p_*Patescibacteria* | 0.2 | O/E > 5.0 | 3.0-5.0 | 1.1-3.0 | < 1.1 |
| c_*Gammaproteobacteria* | 0.1 | E/O > 50 | 5.0-50 | 1.4-5.0 | < 1.4 |
| p_*Nitrospirota* | 0.2 | O/E > 5.0 | 2.0-5.0 | 1.1-2.0 | < 1.1 |
| o_*Burkholderiales* | 0.1 | E/O > 5.0 | 3.0-5.0 | 1.5-3.0 | < 1.5 |
| c_*Campylobacteria* | 0.1 | E/O > 1000 | 100-1000 | 10-100 | < 10 |
| f_*Comamonadaceae* | 0.1 | E/O > 10.0 | 5.0-10 | 3.0-5.0 | < 3.0 |
| f_*Sulfurimonadaceae* | 0.1 | E/O > 1000 | 100-1000 | 30-100 | < 30 |

O: Observation value; E: Expectation value.

Table S10 The identified accurate rate and error rate of groundwater samples suffered by anthropogenic interferences for each indicator and GMIC based on baseline microbial data at a national scale.

| Indicator | Alarm Value | China | | Beijing region | | Xinjiang region | |
| --- | --- | --- | --- | --- | --- | --- | --- |
|  |  | Accurate rate | Error rate | Accurate rate | Error rate | Accurate rate | Error rate |
| Phylogenetic diversity | O/E ≥ 1.2 | 0.523 | 0.309 | 0.583 | 0.323 | 0.714 | 0.250 |
| p_*Patescibacteria* | O/E ≥ 1.1 | 0.492 | 0.266 | 0.417 | 0.323 | 0.286 | 0.292 |
| c_*Gammaproteobacteria* | E/O ≥ 1.4 | 0.554 | 0.349 | 0.667 | 0.290 | 0.143 | 0.083 |
| p_*Nitrospirota* | O/E ≥ 1.1 | 0.423 | 0.163 | 0.667 | 0.161 | 0.286 | 0.167 |
| o_*Burkholderiales* | E/O ≥ 1.5 | 0.508 | 0.382 | 0.833 | 0.323 | 0.286 | 0.292 |
| c_*Campylobacteria* | E/O ≥ 10 | 0.631 | 0.476 | 0.833 | 0.323 | 0.571 | 0.542 |
| f_*Comamonadaceae* | E/O ≥ 3 | 0.515 | 0.287 | 0.750 | 0.194 | 0.286 | 0.292 |
| f_*Sulfurimonadaceae* | E/O ≥ 30 | 0.538 | 0.429 | 0.833 | 0.452 | 0.429 | 0.375 |
| GMIC | ≥1.0 | 0.623 | 0.194 | 0.750 | 0.161 | 0.429 | 0.125 |

O: Observation value; E: Expectation value.

Table S11 The weight and score of each indicator for GMIC based on baseline microbial data at regional scale.

| Region | Indicator | Weight | Score | | | |
| --- | --- | --- | --- | --- | --- | --- |
|  |  |  | 3 | 2 | 1 | 0 |
| Beijing | Taxonomic diversity | 0.1 | O/E > 1.5 | 1.3-1.5 | 1.1-1.2 | < 1.1 |
|  | c_*Campylobacteria* | 0.2 | E/O > 1000 | 500-1000 | 100-500 | < 100 |
|  | o_*Burkholderiales* | 0.1 | E/O > 10 | 5.0-10 | 2.5-5 | < 2.5 |
|  | f_*Sulfurimonadaceae* | 0.1 | E/O > 5000 | 1000-5000 | 100-1000 | < 100 |
|  | f_*Comamonadaceae* | 0.1 | E/O > 50 | 10-50 | 5.0-10 | < 5.0 |
|  | g_*Sulfuricurvum* | 0.1 | E/O > 5000 | 1000-5000 | 300-1000 | < 300 |
|  | p_*Nitrospirota* | 0.1 | O/E > 5 | 2.0-5.0 | 1.1-2.0 | < 1.1 |
|  | g_*Hydrogenophaga* | 0.1 | E/O > 100 | 50-100 | 30-50 | < 30 |
|  | g_*Flavobacterium* | 0.1 | E/O > 1000 | 100-1000 | 30-100 | < 30 |
| Xinjiang | Phylogenetic diversity | 0.1 | O/E > 2.0 | 1.5-2.0 | 1.2-1.5 | < 1.2 |
|  | c_*Gammaproteobacteria* | 0.1 | O/E > 2.0 | 1.5-2.0 | 1.3-1.5 | < 1.3 |
|  | g_*Acidovorax* | 0.1 | O/E > 2.0 | 1.5-2.0 | 1.1-15 | < 1.1 |
|  | c_*Acidimicrobiia* | 0.1 | E/O > 100 | 10-100 | 5.0-10 | < 5.0 |
|  | f_*Vicinamibacteraceae* | 0.1 | E/O > 50 | 10-50 | 4.0-10 | < 4.0 |
|  | p_*Gemmatimonadota* | 0.1 | E/O > 50 | 10-50 | 3.0-10 | < 3.0 |
|  | g_*Rhodobacter* | 0.1 | E/O > 50 | 10-50 | 5.0-10 | < 5.0 |
|  | f_*Halomonadaceae* | 0.1 | E/O > 1000 | 500-1000 | 100-500 | < 100 |
|  | f_*Bacillaceae* | 0.1 | E/O > 100 | 50-100 | 10-50 | < 10 |
|  | f_*Nitrosomonadaceae* | 0.1 | E/O > 500 | 100-500 | 10-100 | < 10 |

O: Observation value; E: Expectation value.
