# Supplementary material for: May microbial ecological baseline exist in continental groundwater?
Source: Microbiome. 2023 Jul 19;11:152. doi: 10.1186/s40168-023-01572-4 (PMC10355068; doi:10.1186/s40168-023-01572-4)
Supplement: Supplementary file 3 — Additional file 2: Fig. S1. Taxonomic percentage of sequences (a) and OTUs (b) for the overall bacterial communities at phylum level. Fig. S2. a, Rank-abundance curves for bacterial community; b, Percentage numbers and relative abundance of dominant OTUs (average abundance > 0.01%) in groundwater sampled from different types of wells. Fig. S3. Longitudinal distributions of microbial taxonomic and phylogenetic diversity in groundwater. Red solid and black dashed lines show polynomial and linear fits based on ordinary least-square regression analysis, with the shaded area representing 95% confidence intervals. Adjusted R2 of the polynomial fits and Pearson’s r of the linear fits are provided. Fig. S4. Violin plot comparing microbial diversity across seven geo-environmental zones in phreatic water of varying burial depth ranges. Fig. S5. Distance-decay curves showing the relationship between geographic distance and community similarity. Red and blue lines denote the least-square linear regressions between geo-environmental zones and within the same geo-environmental zone. Slope and P values (one-sided) for regression slopes are stated. Fig. S6. Depth stratification of microbial communities in pristine groundwater. a, Alpha-diversity variation with well depth in newly constructed wells (n = 733), confined water (n = 229), and phreatic water (n = 504). Black and red lines show linearand polynomial regressions, with shaded representing 95% confidence intervals. b, Non-metric multidimensional scaling (NMDS) analysis based on Bray-Curtis similarity showing compositional variation with well depth in phreatic water. The color gradient denotes the well depth of each sample. c, Boxplot of community similarity in phreatic water for three well depth ranges. Asterisks denote the significance of correlations (***P <0.001). Fig. S7. Vertical distributions of microbial diversity inphreatic water under varying geo-environments. Adjusted R2 of the polynomial fits are provided. Fig. S8. [file 40168_2023_1572_MOESM2_ESM.docx]

**Additional file 2**

**May microbial ecological baseline exist in continental groundwater?**

**Authors**: Sining Zhong^1,2,3^, Shungui Zhou^3^, Shufeng Liu^1^, Jiawen Wang^1^, Chenyuan Dang^1^, Qian Chen^1,4^, Jinyun Hu^1^, Shanqing Yang^1^, Chunfang Deng^1^, Wenpeng Li^5^, Juan Liu^1^, Alistair G.L. Borthwick^6, 7^, Jinren Ni^1,2^*

**Author affiliations**:

^1^College of Environmental Sciences and Engineering, Peking University; Key Laboratory of Water and Sediment Sciences, Ministry of Education, Beijing 100871, P. R. China

^2^State Environmental Protection Key Laboratory of All Material Fluxes in River Ecosystems, Beijing 100871, P. R. China

^3^Fujian Agriculture and Forestry University, College of Resources and Environment, Fujian Provincial Key Laboratory of Soil Environment Health and Regulation, Fuzhou 350002, P. R. China

^4^State Key Laboratory of Plateau Ecology and Agriculture, Qinghai University, Xining 810016, P. R. China

^5^Center for Groundwater Monitoring, China Institute of Geo-environmental Monitoring, Beijing 100081, P. R. China

^6^School of Engineering, Computing and Mathematics, University of Plymouth, Drake Circus, Plymouth PL8 4AA, UK.

***Corresponding author:** Jinren Ni

Postal address: Peking University, No. 5 Yiheyuan Road, Beijing 100871, P. R. China

Telephone number: +86-10-62751185

E-mail address: jinrenni@pku.edu.cn


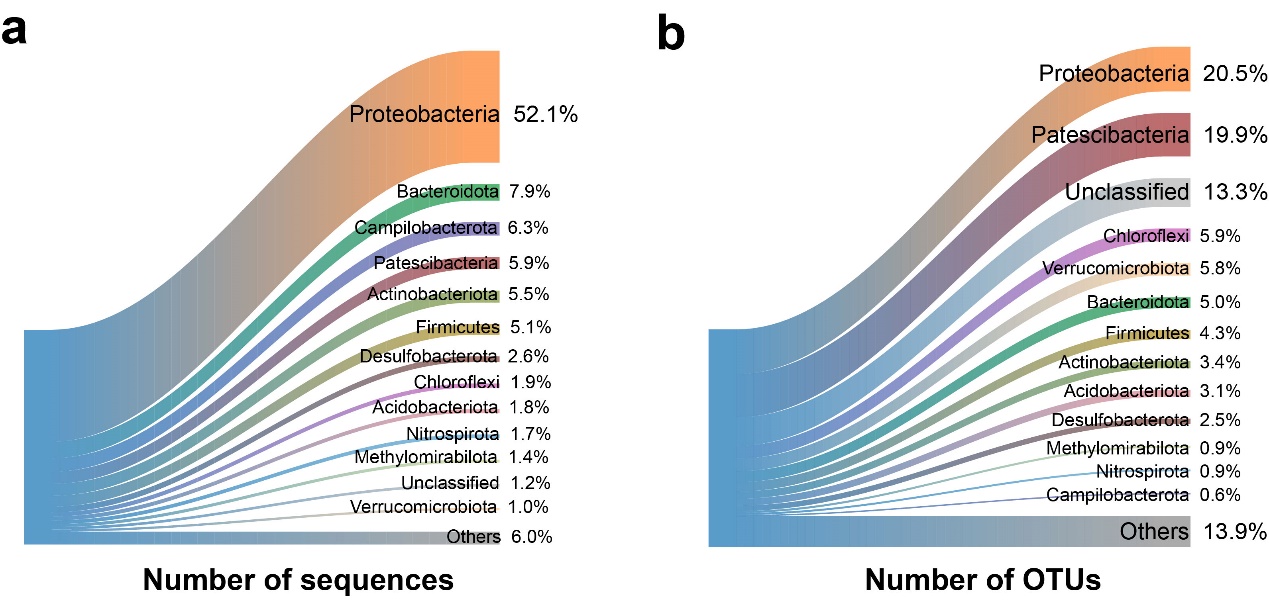


**Fig. S1** Taxonomic percentage of sequences (**a**) and OTUs (**b**) for the overall bacterial communities at phylum level.


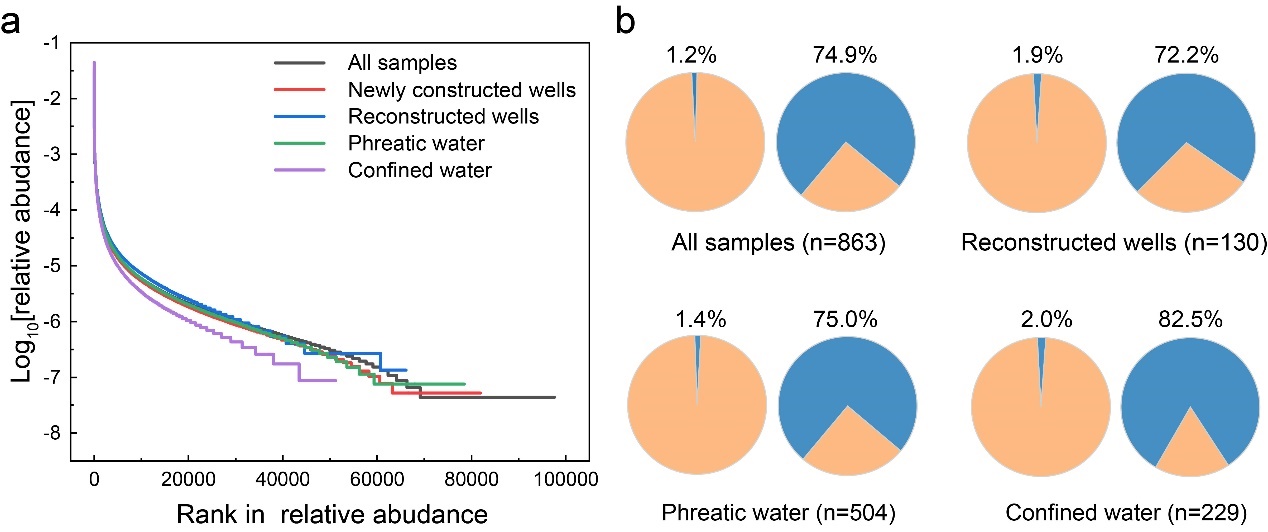


**Fig. S2 a,** Rank-abundance curves for bacterial community; **b,** Percentage numbers and relative abundance of dominant OTUs (average abundance > 0. 01%) in groundwater sampled from different types of wells.


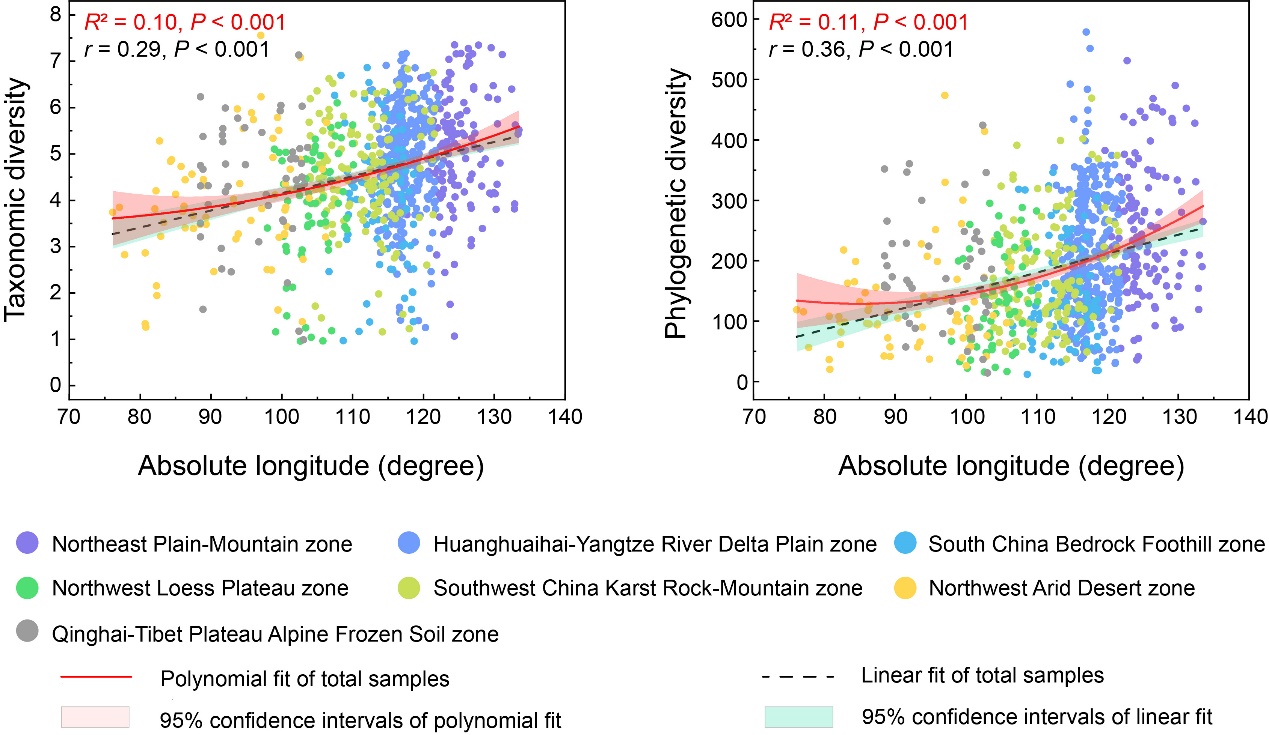


**Fig. S3** Longitudinal distributions of microbial taxonomic and phylogenetic diversity in groundwater. Red solid and black dashed lines show polynomial and linear fits based on ordinary least square regression analysis, with the shaded area representing 95% confidence intervals. Adjusted *R*^2^ of the polynomial fits and Pearson’s *r* of the linear fits are provided.


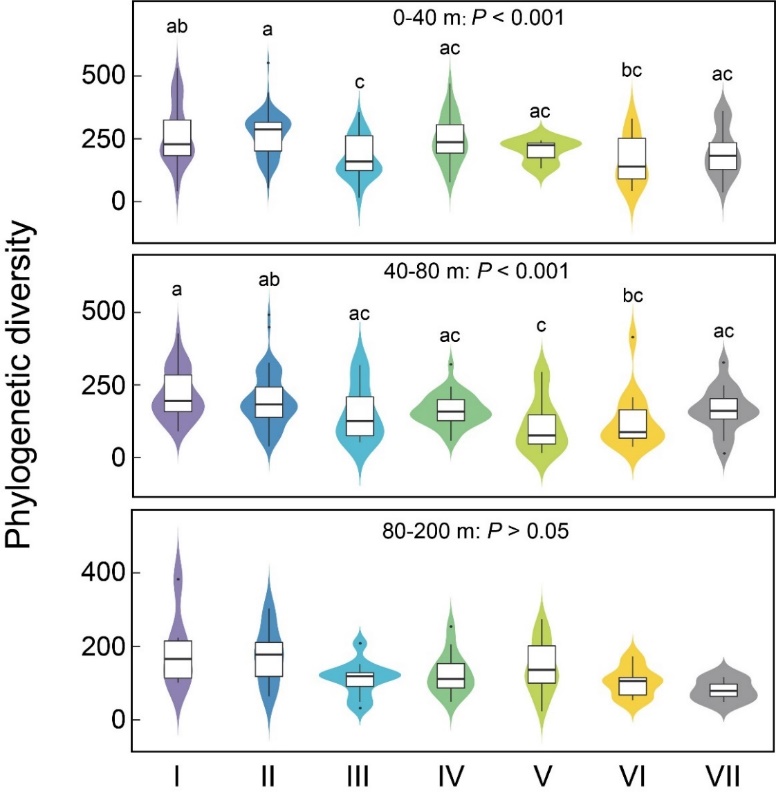


**Fig. S4** Violin plot comparing microbial diversity across seven geo-environmental zones in phreatic water of varying burial depth ranges.


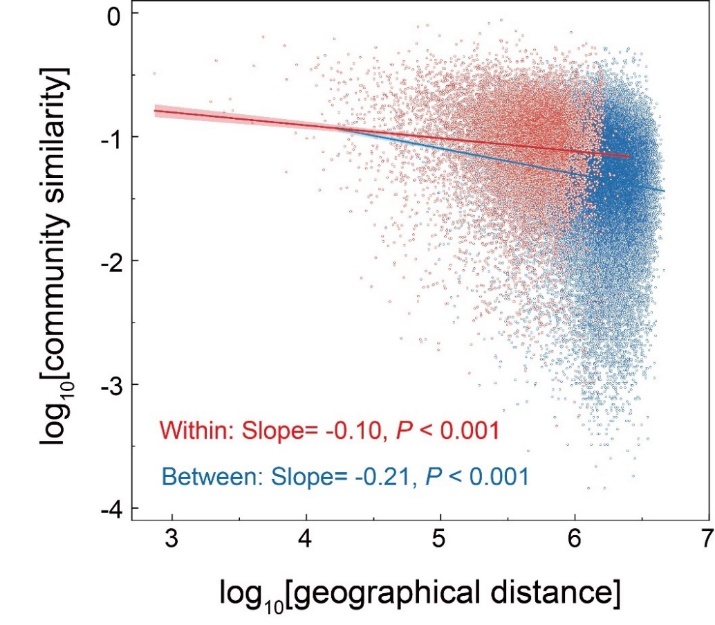


**Fig. S5** Distance-decay curves showing the relationship between geographic distance and community similarity. Red and blue lines denote the least-squares linear regressions between geo-environmental zones and within the same geo-environmental zone. Slope and P values (one-sided) for regression slopes are stated.


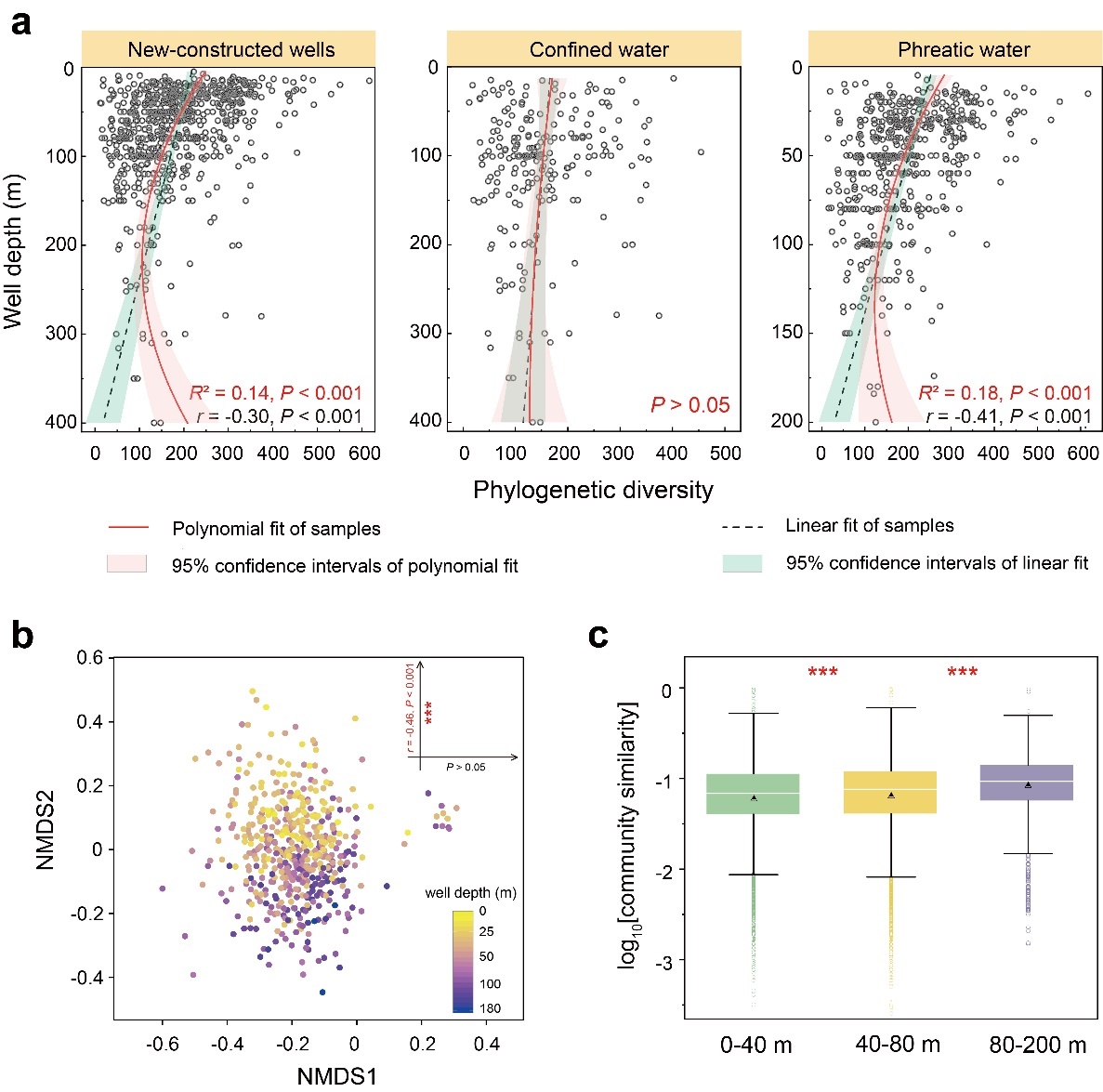


**Fig. S6** **Depth stratification of microbial communities in pristine groundwater. a**, Alpha-diversity variation with well depth in newly constructed wells (n = 733), confined water (n = 229), and phreatic water (n = 504). Black and red lines show linear and polynomial regressions, with shaded areas representing 95% confidence intervals. **b**, Non-metric multidimensional scaling (NMDS) analysis based on Bray-Curtis similarity showing compositional variation with well depth in phreatic water. The color gradient denotes the well depth of each sample. **c**, Boxplot of community similarity in phreatic water for three well depth ranges. Asterisks denote the significance of correlations (****P* < 0.001).

**
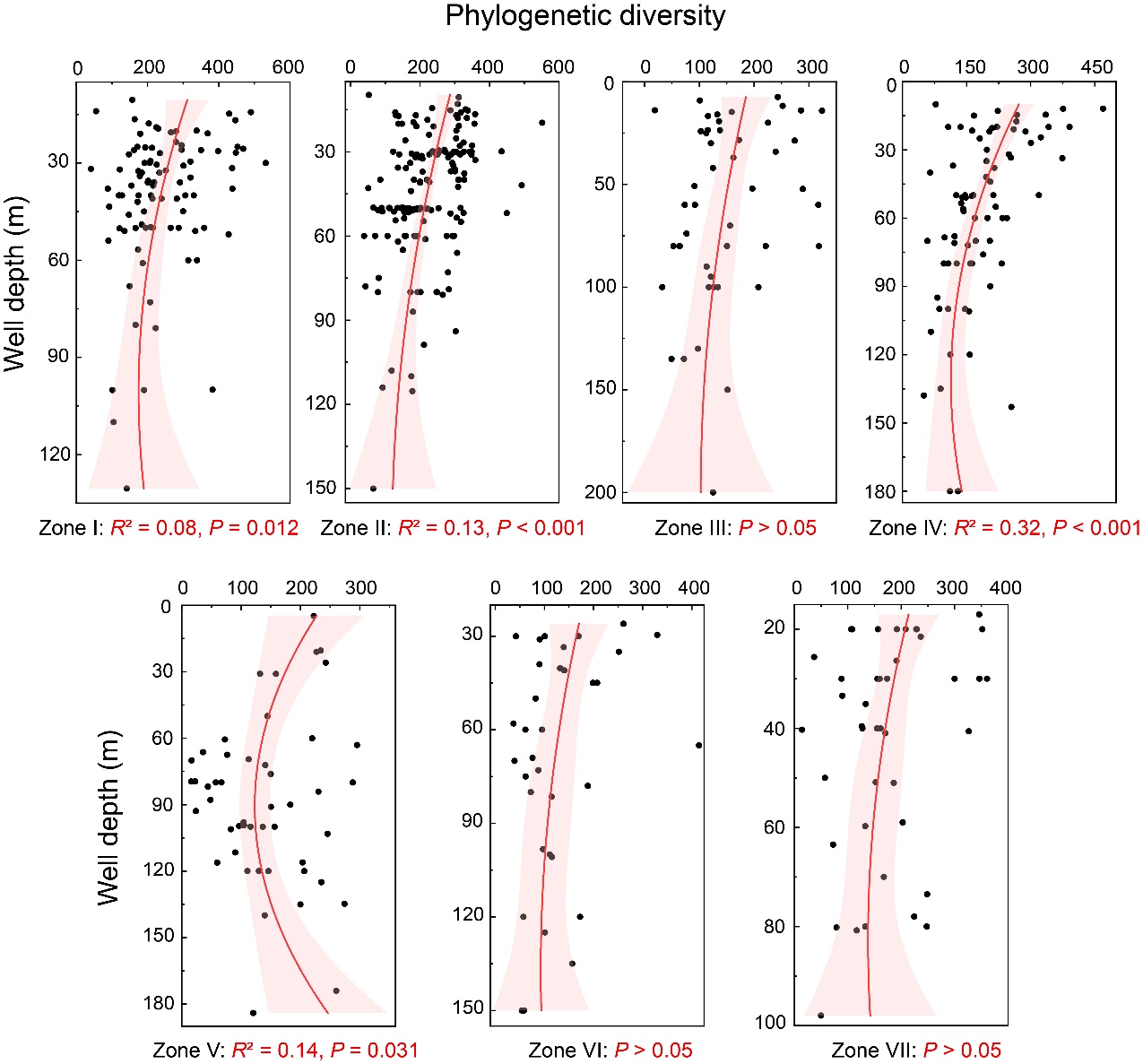
**

**Fig. S7** Vertical distributions of microbial diversity in phreatic water under varying geo-environments. Adjusted *R*^2^ of the polynomial fits are provided.


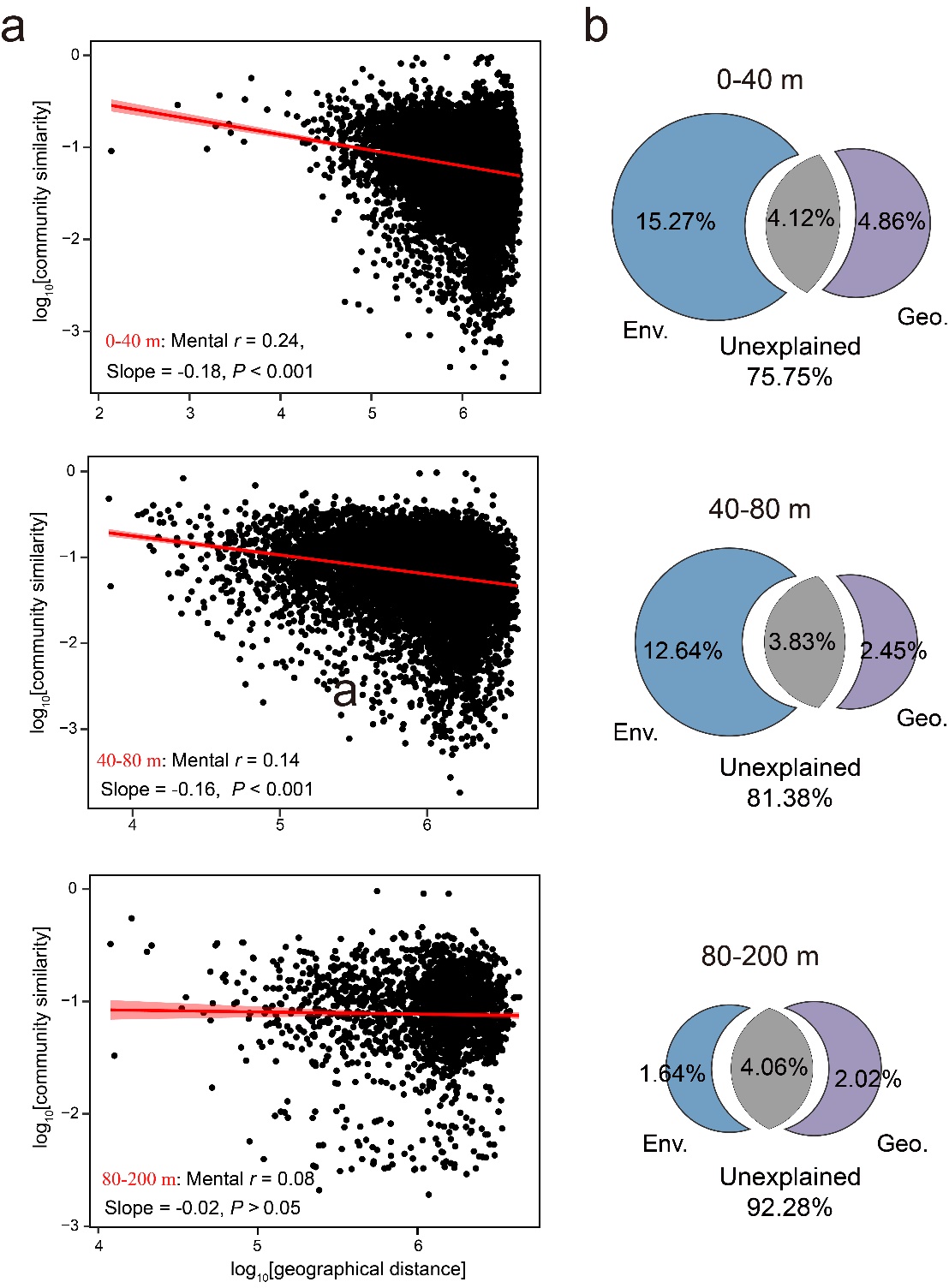


**Fig. S8** **a**, Distance-decay relationships (DDRs) showing community similarity against geographic distance between sampling sites in phreatic water of three well depth ranges. Red lines denote ordinary least-squares linear regressions, with the shaded area representing 95% confidence intervals. Slope of DDRs, mantel Spearman correlations (*r*), and probabilities (P) are listed in the legends. **b**, Variance partition analysis showing relative contributions of environmental (Env.) and geographical (Geo.) factors and their combined effect on community variations based on Bray-Curtis similarity.


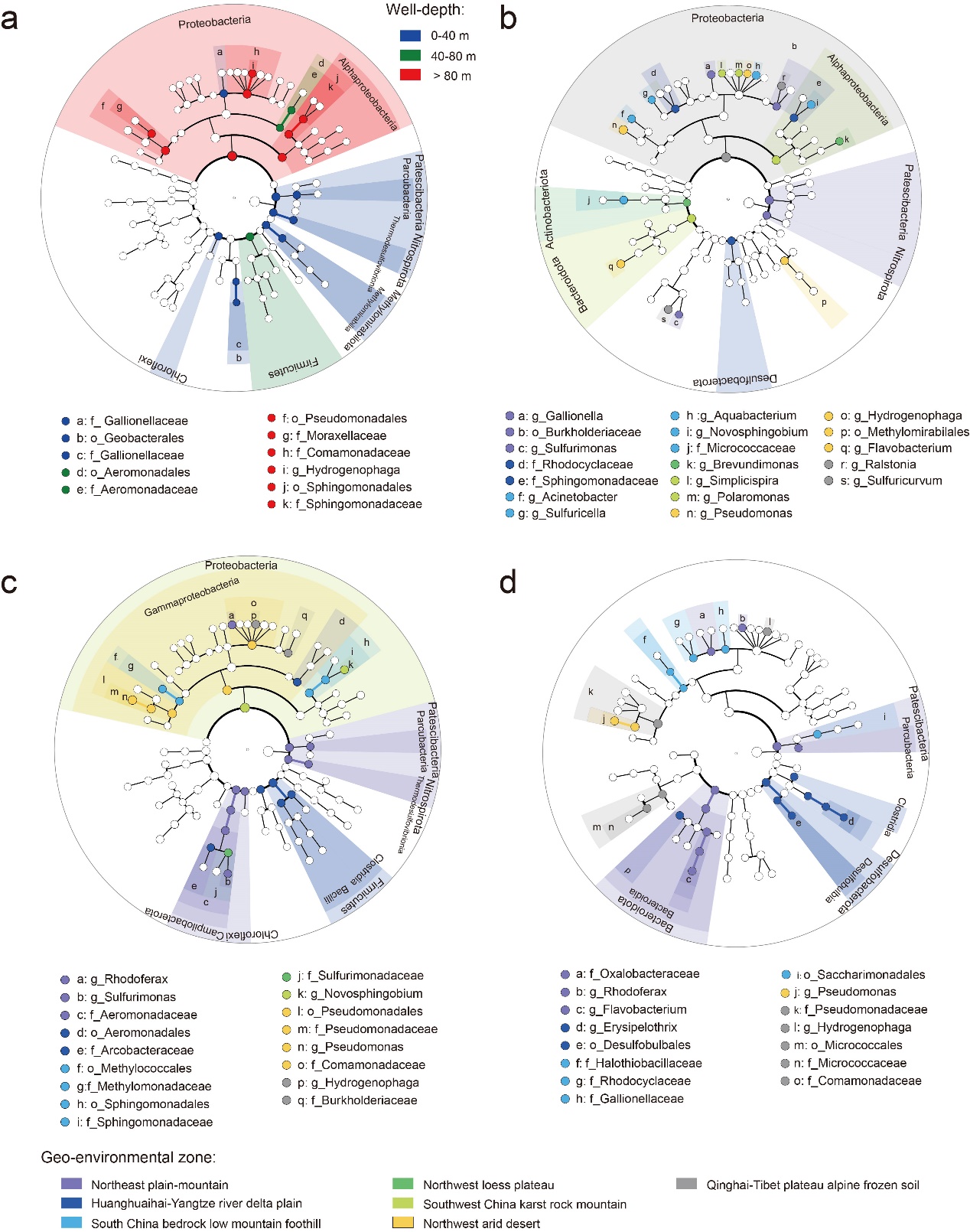


**Fig. S9** LEfSe cladogram of microbial community obtained for varying well-depth ranges (a), geo-environmental zones at shallower (b), [medium](javascript:;) (c) and deeper (d) phreatic water. All detected taxa with average relative abundance≥0.5% were assigned to domain (innermost), phylum, class, order, family, and genus (outermost). Differentially abundant taxa (biomarkers) are colored according to their most abundant regions.


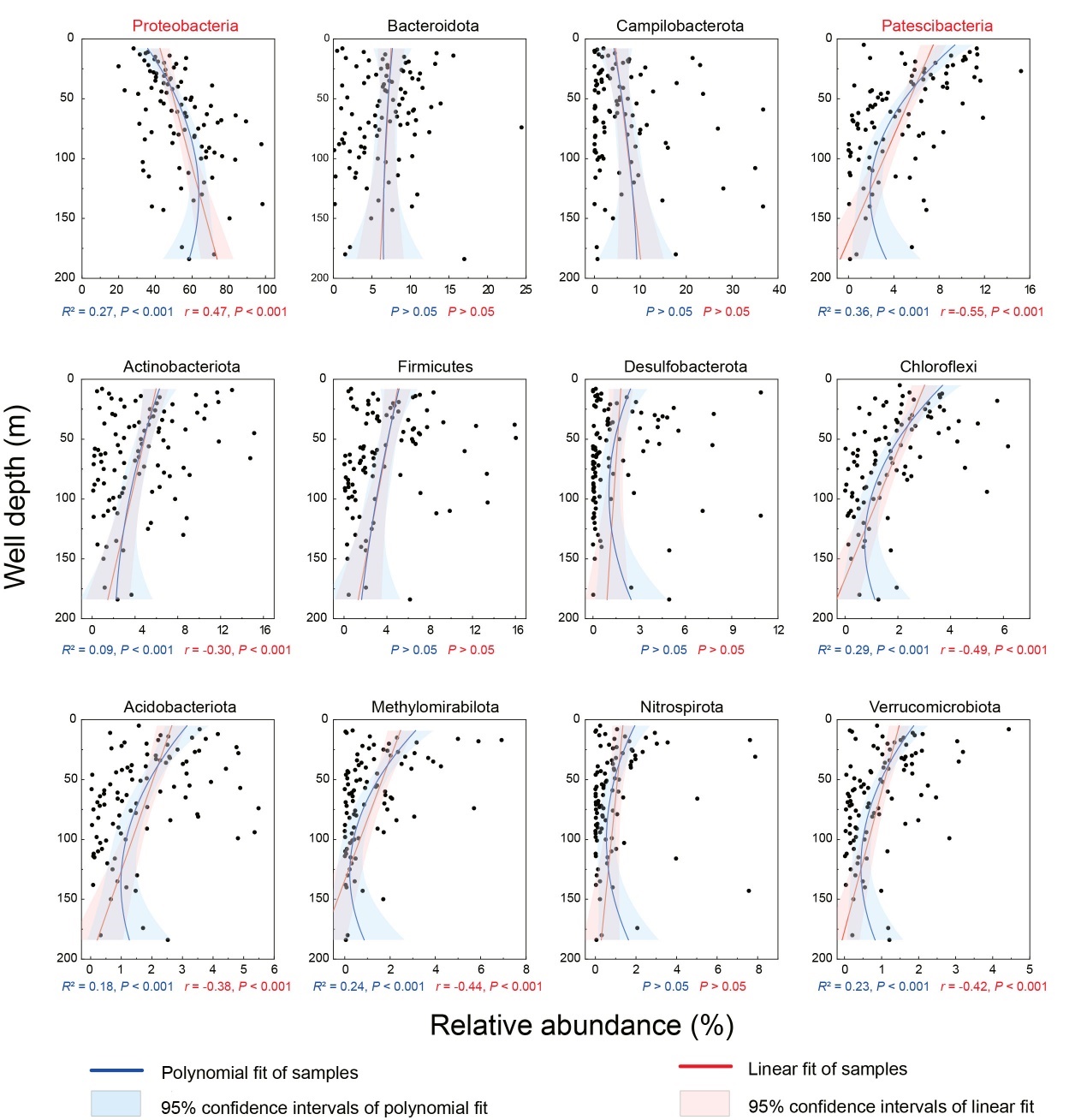


**Fig. S10** Variation in relative abundance of dominant phyla with well depth in phreatic water. Red and blue lines show linear and polynomial fits based on ordinary least square regression, with the shaded areas representing 95% confidence intervals. Adjusted *R*^2^ of the polynomial fits and Pearson’s *r* of the linear fits are provided. Variation in relative abundance of dominant phyla with well depth in phreatic water


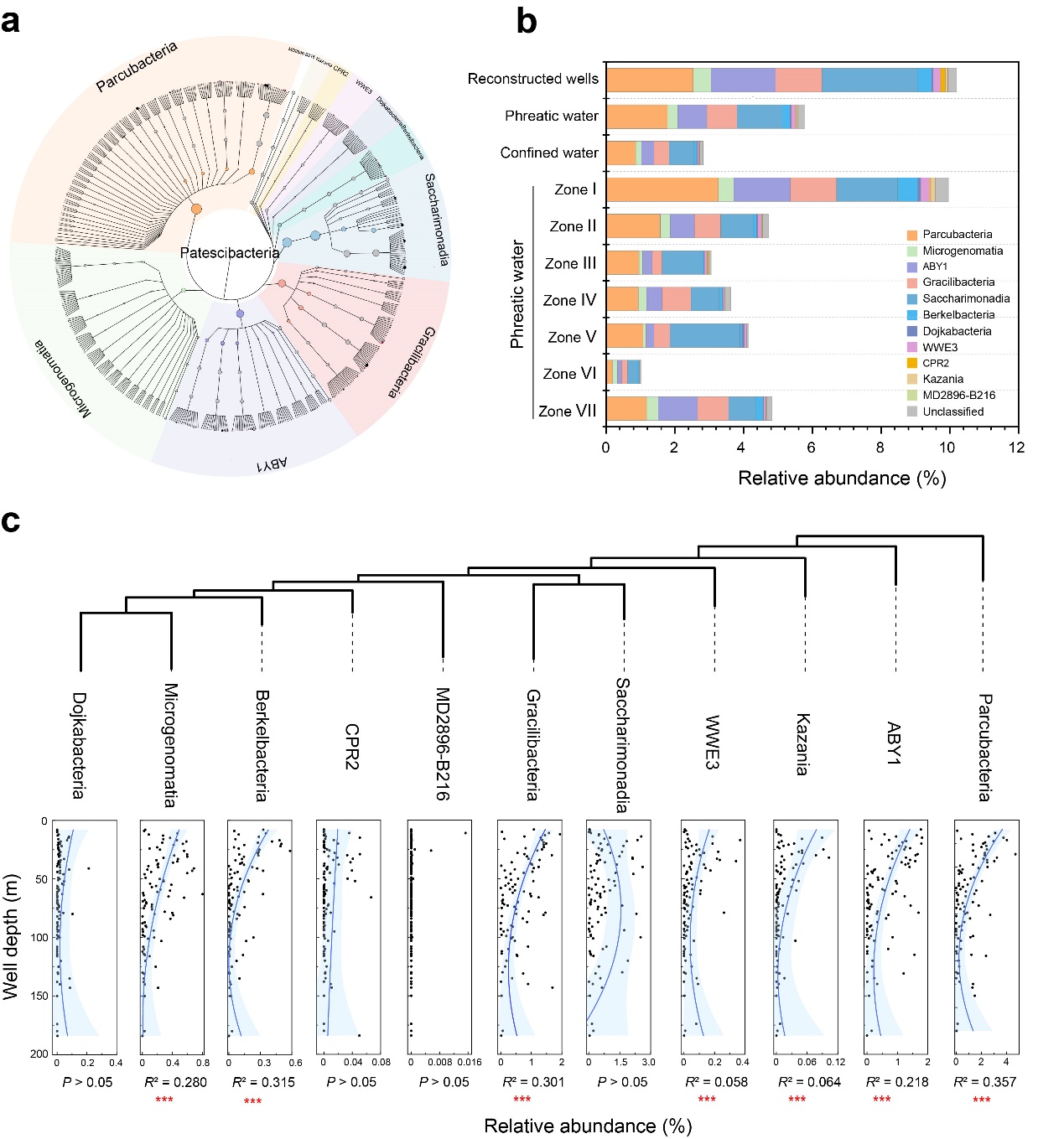


**Fig. S11 Composition and distribution of Patescibacteria in groundwater. a**, Taxonomic tree of identified Patescibacteria OTUs in groundwater. The color of circles indicates taxonomic information of each OTU, and the size of circles is proportional to the relative abundance. The grey circles represent the OTUs with no affiliation at the corresponding taxonomic level. **b**, Average relative abundance of Patescibacteria classes in varying types of wells and geo-environmental zones. **c,** Variation of the relative abundance of Patescibacteria classes with well depth in phreatic water. The blue lines show the polynomial fit based on ordinary least squares regression, with the shaded areas representing 95% confidence intervals. *0.01 < *P* < 0.05, **0.001 < *P* < 0.01 and ****P* < 0.001.

**
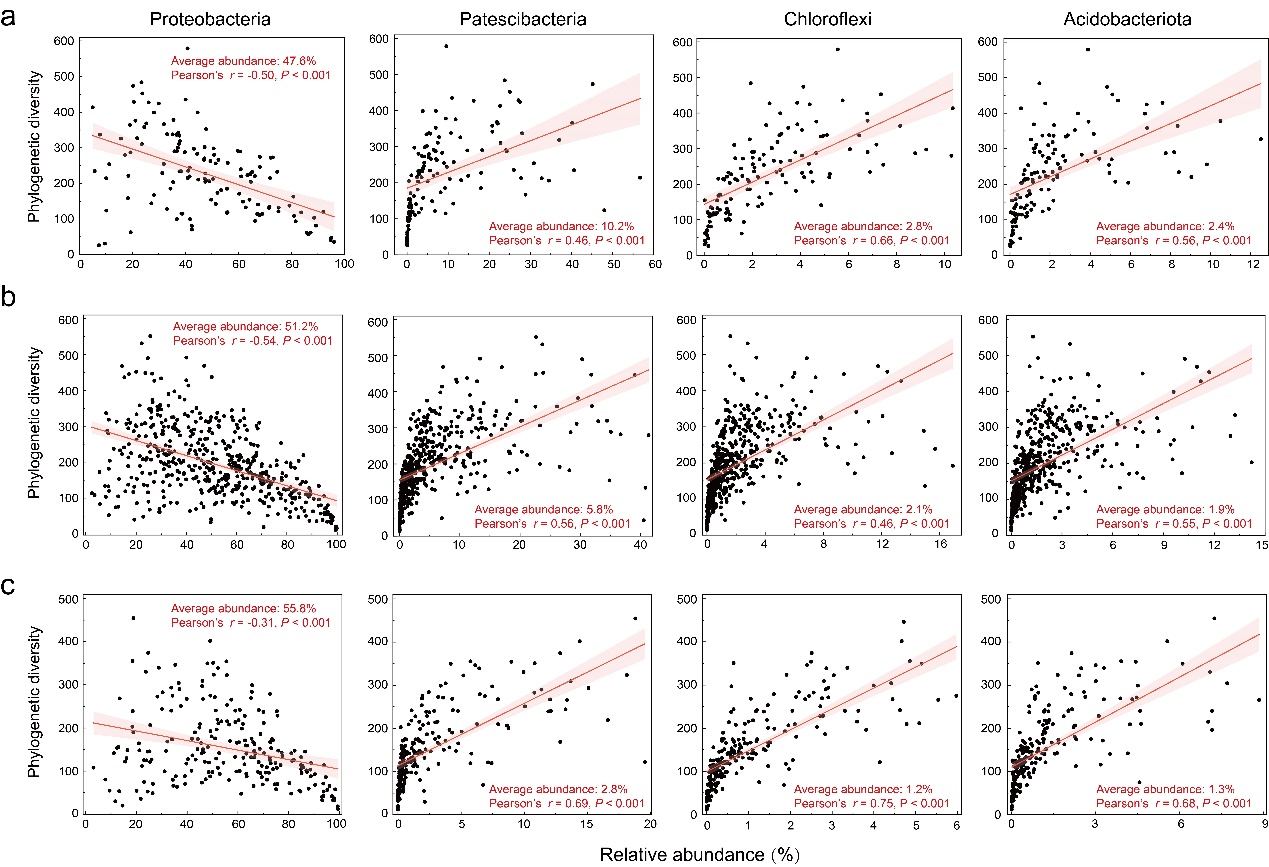
**

**Fig. S12** Relationship between microbial diversity and relative abundance of Proteobacteria, Parcubacteria, Chloroflexi, and Verrucomicrobiota in reconstructed wells (**a**), phreatic water (**b**), and confined water (**c**). Red lines indicate ordinary least square linear regressions across all samples in each habitat. Shaded areas represent 95% confidence intervals. Adjusted Pearson correlations (*r*) of the linear fits are provided.


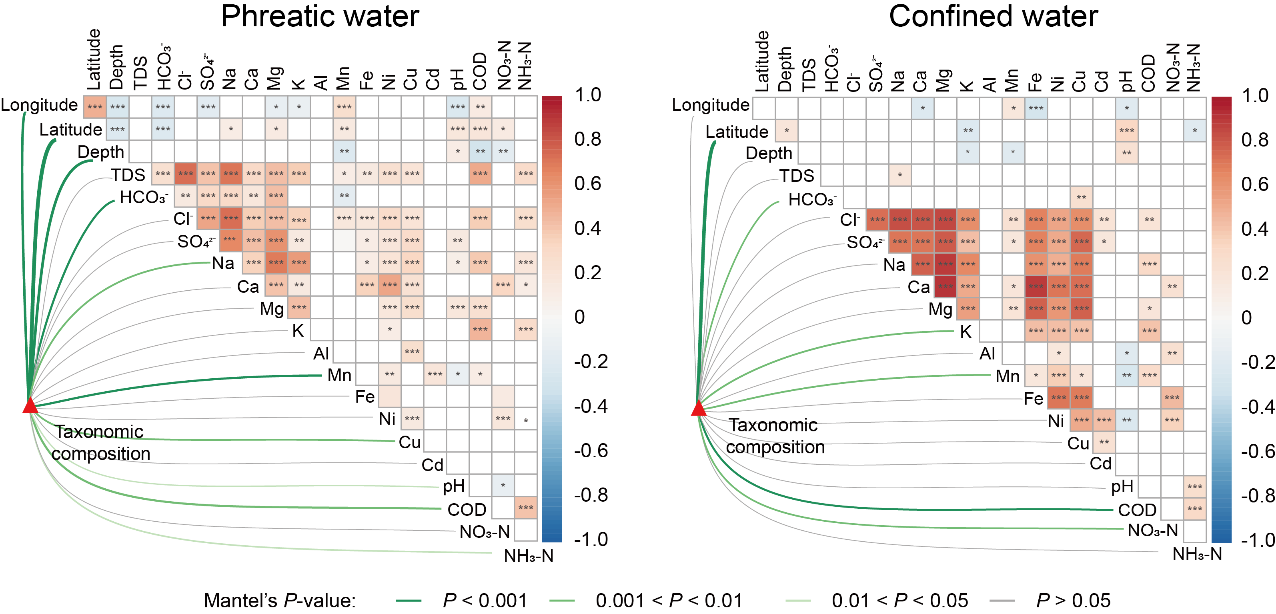
 **Fig. S13** Ecological drivers of microbial community in phreatic and confined water. Pairwise Spearman correlation coefficients between environmental parameters are indicated by the color gradient. Asterisks denote the significance of correlations (*0.01 < *P* < 0.05, **0.001 < *P* < 0.01, and ****P* < 0.001). Taxonomic composition (based on Bray-Curtis distance) was related to each environmental parameter (based on Euclidean distance) by Mantel tests. Edge width corresponds to Mantel *r* statistics, and edge color denotes statistical significance.

**
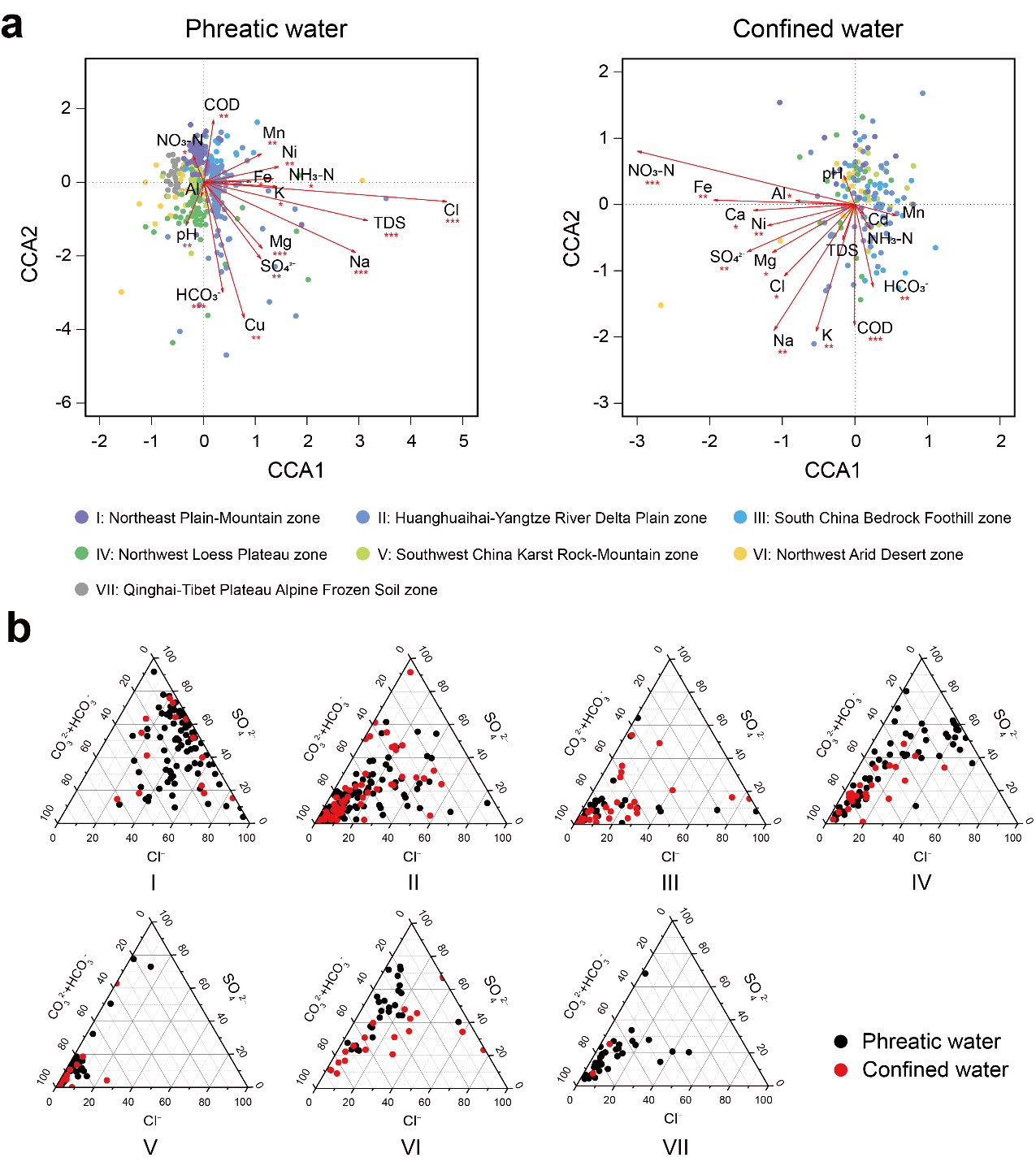
 Fig. S14** **a,** Constrained correspondence analyses (CCA) reveal environmental parameters governing the distribution of microbial communities in phreatic and confined water. **b,** The panels show respective geochemical signatures as percentages of chloride (Cl^−^), sulfate (SO_4_^2−^), and the sum of carbonate (CO_3_^2−^) and bicarbonate (HCO_3_^−^) ions in phreatic and confined water for the seven geo-environments in China.

**
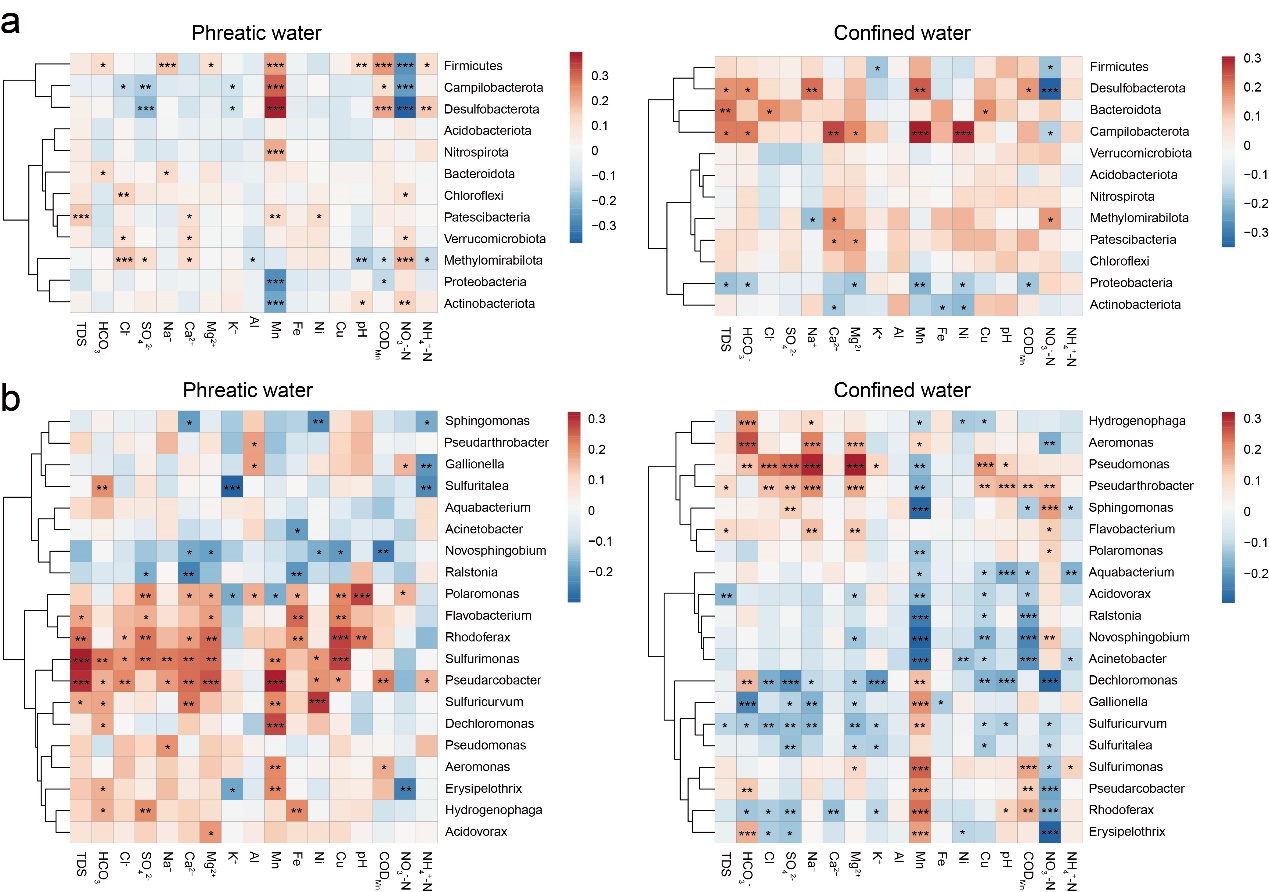
**

**Fig. S15** Cluster heatmap analysis demonstrating that microbial dominant taxa (average relative abundance > 1%) at phylum **(a)** and genus **(b)** level display abundance patterns corresponding to geochemical parameters.


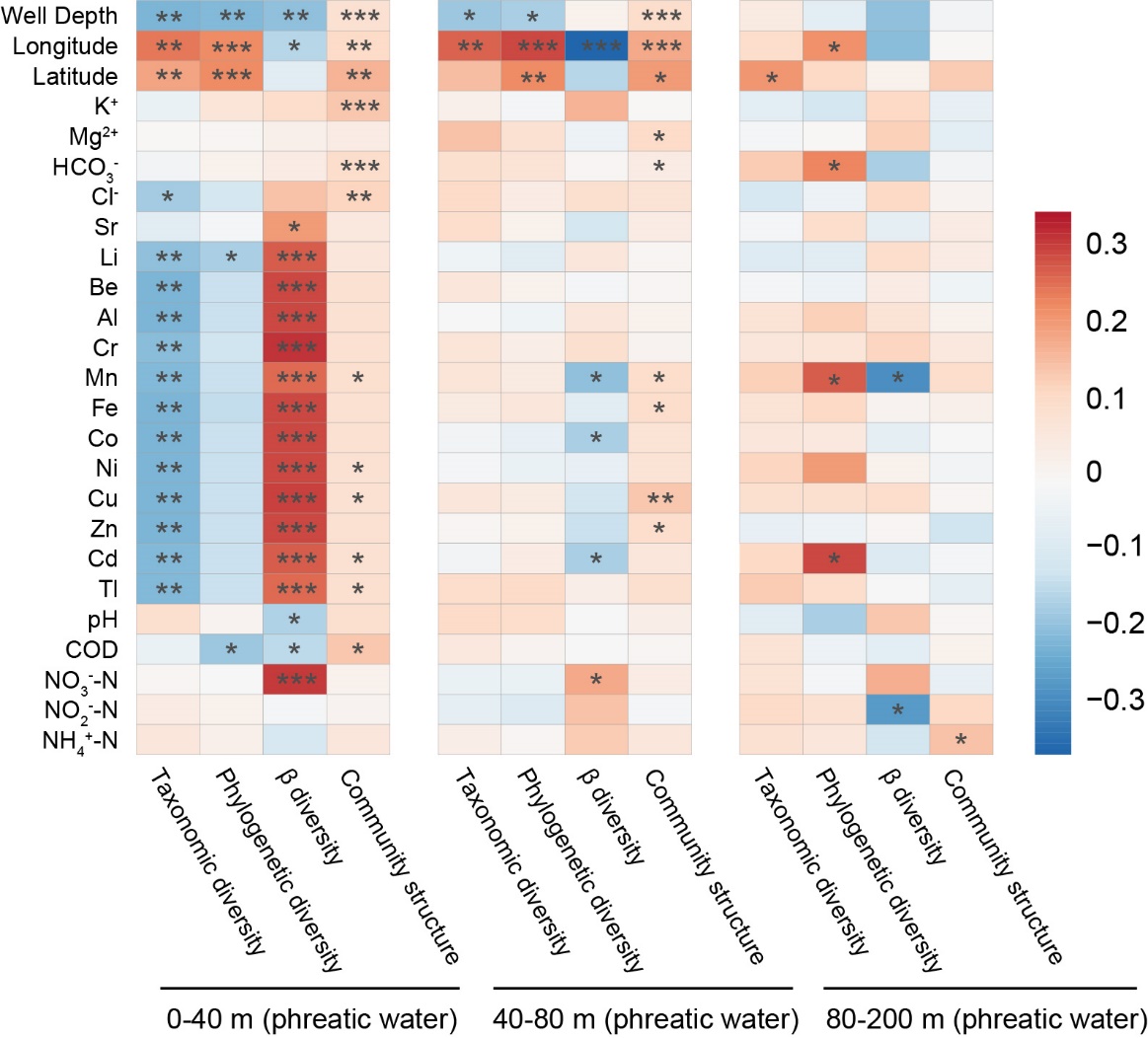


**Fig. S16** Heatmap showing Pearson correlation between main geographical and environmental factors and the diversity and structure of microbial communities in phreatic water for different well depth groups. Asterisks denote the significance of correlations (*0.01 < *P* < 0.05, **0.001 < *P* < 0.01, and ****P* < 0.001).

**
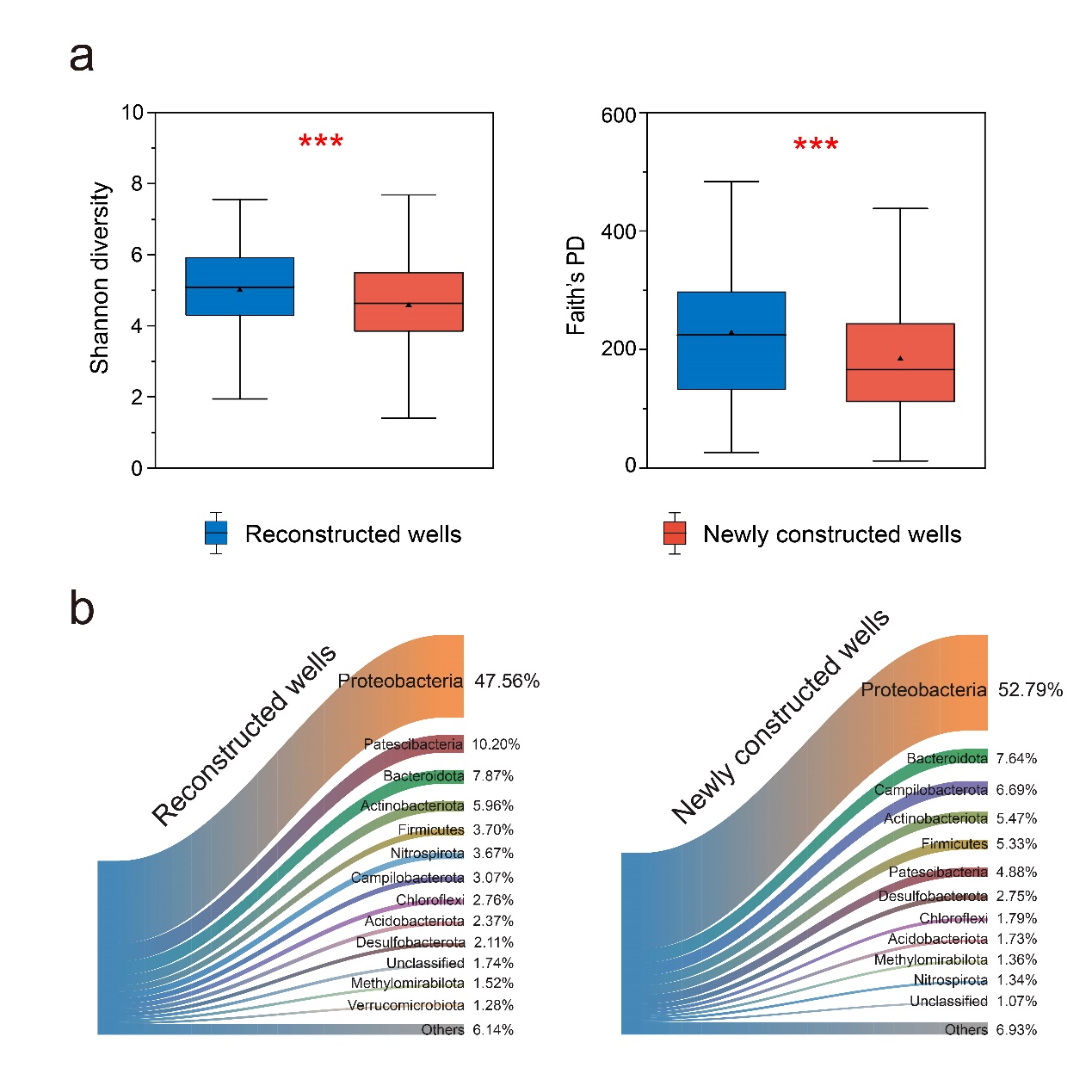
**

**Fig. S17** Comparison of diversity and composition of microbial communities between reconstructed and newly constructed wells. **a,** Boxplots showing that both taxonomic and phylogenetic diversities in reconstructed wells are significantly higher than in newly constructed wells. The hinges show the 25th, 50th and 75th percentiles. **b,** Sankey diagrams showing the relative abundances of dominant phyla (> 1%) in reconstructed and newly constructed wells.


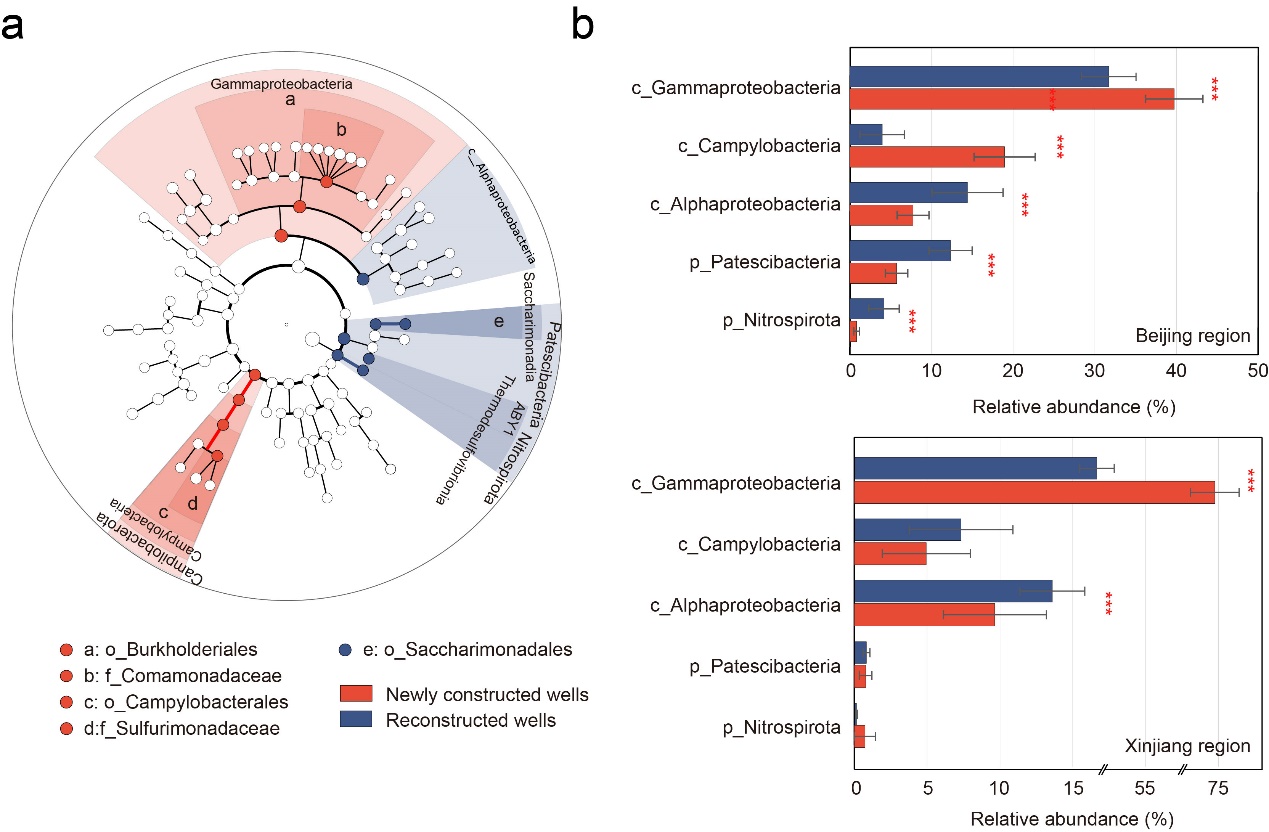
 **Fig. S18 a,** LEfSe analysis identifying the groundwater biomarker of reconstructed (blue) and newly constructed wells (red). All detected taxa (relative abundance ≥ 0.5%) are assigned to domain (innermost), phylum, class, order, family, and genus (outermost). Taxa meeting an LDA significant threshold of > 4.0 are selected as most likely to explain differences in microbial communities between reconstructed and newly constructed wells. **b,** Comparison of average relative abundance of biomarkers between reconstructed and newly constructed wells in Beijing and Xinjiang regions.
